# Within-individual precision mapping of brain networks exclusively using task data

**DOI:** 10.1016/j.neuron.2025.08.029

**Published:** 2025-09-26

**Authors:** Jingnan Du, Vaibhav Tripathi, Maxwell L. Elliott, Joanna Ladopoulou, Wendy Sun, Mark C. Eldaief, Randy L. Buckner

**Affiliations:** 1 Department of Psychology, Center for Brain Science, Harvard University, Cambridge, MA 02138, USA; 2 Department of Psychiatry, Massachusetts General Hospital, Charlestown, MA 02129, USA; 3 Athinoula A. Martinos Center for Biomedical Imaging, Massachusetts General Hospital, Charlestown, MA 02129, USA; 4 Lead contact

## Abstract

Precision mapping of brain networks within individuals prevailingly relies on functional connectivity analysis of resting-state data. Here, we explored whether networks can be estimated using only task data. Correlation matrices estimated from task data were similar to those derived from resting-state data. The largest factor affecting similarity was the amount of data. Precision networks estimated from task data showed strong spatial overlap with those derived from resting-state data and predicted the same triple functional dissociation in independent data. To illustrate novel possibilities enabled by the present methods, we mapped the detailed organization of thalamic association zones within individuals by pooling extensive resting-state and task data. We also demonstrated how task data can be used to estimate networks while simultaneously extracting task responses. Broadly, these findings suggest that there is an underlying, stable network architecture that is idiosyncratic to the individual and persists across task states.

## In brief

Du et al. demonstrate that task data alone can map the detailed organization of within-individual brain networks. Applications include reanalysis of existing data, pooling of task and resting-state data to increase statistical power, and studies that solely acquire task data to simultaneously estimate brain networks and extract the evoked response.

## INTRODUCTION

The organization of human brain networks can be estimated by measuring spontaneous correlations of the blood oxygenation level-dependent (BOLD) signal, a technique referred to as functional connectivity MRI (fcMRI; Biswal et al.^1^; see also Fox and Raichle,^2^ Van Dijk et al.,^3^ Deco et al.,^4^ Murphy et al.,^5^ Smith et al.,^6^ Power et al.,^7^ Lv et al.,^8^ Gratton et al.,^9^ and Buckner et al.^10^). fcMRI most often uses data acquired during passive states, such as when participants rest with their eyes closed or visually fixate on a crosshair. Passive data collection was the procedure adopted early in demonstrations of the technique^1,11^ and has remained the mainstay of the field in part because of its simplicity. However, in many instances data have been, or will be, exclusively collected during active task paradigms designed to elicit time-locked functional responses, and those data have been considered generally less useful for network analysis.

Some applications require both passive data and active task data. For example, passively acquired data have been extensively employed to estimate brain networks within the idiosyncratic anatomy of individuals.^12–16^ The networks can then serve as localizers to explore response properties in active task paradigms, including to dissociate functionally distinct side-by-side regions that vary slightly in their positions from one person to the next (e.g., Tobyne et al.,^17^ DiNicola et al.,^18^ Du et al.,^19^ and Edmonds et al.^20^; see also Tripathi et al.^21^ and Tavor et al.^22^). These combination brain mapping experiments often require long (or multiple) sessions given the needed time to separately acquire passive and active task scans.

Here, we explore the possibility of exclusively using task data to precisely map brain networks within individuals. Serving as the foundation for the present work, prior group-based studies have examined functional connectivity estimates from task acquisitions as compared with passive acquisitions. Fair et al.^23^ specifically raised the possibility of regressing task structure from scans collected during active tasks and then conducting functional connectivity analyses on the residualized data (see also Arfanakis et al.^24^). They observed considerable similarity between the correlation patterns derived from task-regressed data and traditional estimates from passive acquisitions, as well as some differences. Follow-up analyses using multiple acquisition strategies and analysis procedures have found similar results.^25–33^ In an extensive set of analyses, Gratton et al.^34^ provided evidence that functional connectivity matrices are dominated by stable individual factors, with only a modest contribution from task demands. These prior studies, taken collectively, suggest that the majority of the region-to-region correlation structure found to be present in resting-state acquisitions is also present in task acquisitions.

However, the portion of the functional correlations sensitive to task states may be sufficient to undermine precision brain mapping (e.g., Geerligs et al.^35^ and Salehi et al.^36^; see also Gratton et al.^9^ and Shirer et al.^37^). In an influential set of explorations, Salehi and colleagues^36^ illustrated that the borders and exact locations of estimated cortical regions vary as a function of the acquisition task, raising concerns that the estimated regions do not reflect stable, biological subdivisions of the cortex. In a critical analysis, they demonstrated that the task acquisition state could be predicted from the obtained parcellation. Such observations have limited the adoption of task data for the purpose of functional connectivity estimates of brain networks. Nevertheless, even within the analyses of Salehi et al.,^36^ there was a high degree of correspondence between parcellation estimates between tasks vs. within tasks (see their Figure 3D). This suggests that while there is agreement that the task acquisition state affects a portion of the correlation structure of the data, it is an open question whether the task contribution can be sufficiently minimized and the commonalities extracted to estimate stable networks within individuals.

Here, we demonstrate that, despite historical reservations, extracting the stable portions of the functional connectivity matrix—when combined with proper precision modeling of networks—can yield robust and valid estimates of networks within individuals exclusively using task data. This finding significantly expands the possibilities for how we conduct and analyze neuroimaging data, and we demonstrate several novel applications. Specifically, we found that network estimates derived from task data are remarkably similar to those obtained from traditional resting-state fixation (FIX) acquisitions, including the preservation of fine-grained, individual-specific network features. This indicates that the stable portions of the correlation structure within task data are sufficient to estimate precision networks and localize regions within individuals. We further show that networks estimated from task-based data can effectively predict functional specializations across multiple higher-order cognitive domains in independent task datasets; that detailed organization in deep brain structures, including the thalamus, can be estimated by aggregating task and resting-state data—achieving the necessary level of signal averaging; and finally, that the same task data can simultaneously provide both within-individual network estimates and region-level functional response quantification. These findings open the door to novel discoveries in previously collected datasets and lay the groundwork for incorporating innovative experimental designs into future studies.

## RESULTS

### Functional correlation matrices derived from task data are highly similar to those derived from resting-state acquisitions

Functional connectivity analyses use the region-to-region correlation matrix of the fMRI time series to estimate networks. Our first results examined the similarity between raw correlation matrices derived from task-regressed data (motor [MOT] and episodic projection [EPRJ]) as compared with traditional resting-state FIX data. To fully contextualize the correspondence, correlation matrices from independent datasets acquired in the same manner were also examined (e.g., the FIX data were split into independent FIX1 and FIX2 datasets). Four findings were observed (Figure S1A).

First, the similarity of the correlation matrices estimated from task-regressed data to the resting-state FIX data was almost as high as any of the data types to themselves. Some variance was related to the task acquisition state, but it was observed to be a relatively small portion of the variance when considering the correlation of same-type data acquisitions to themselves. For example, for the case of 4 runs of data per dataset, the matrices within the same acquisition type correlated with each other with a range of 0.79–0.81 (FIX1 vs. FIX2, MOT1 vs. MOT2, and EPRJ1 vs. EPRJ2). The correlations between distinct acquisition types ranged from 0.66 (EPRJ1 vs. MOT1) to 0.75 (FIX1 vs. EPRJ1). That is, the vast amount of variance in the correlation matrices was similar regardless of the task performed at the time of data acquisition. This result was found whether the task was a low-level sensorimotor task (MOT) or a higher-order task requiring participants to make challenging judgments during extended epochs (EPRJ).

Additional analyses confirmed this finding. When comparing the similarity of the correlation matrices across different individuals and acquisition types, we observed a low similarity, with a mean correlation of 0.32. Networks from the same individual but different acquisition types were more similar to each other, with a mean correlation of 0.71, demonstrating a large influence of within-individual analyses (Figure S1E). Correlation matrix similarity from the same individual and acquisition type was slightly higher than the similarity between different acquisition types within the same individual, with a mean correlation of 0.80, demonstrating a relatively small task effect on the correlation matrix similarity. The observed similarity effect for individuals and acquisition types is consistent with previous findings (see Figure 3B from Gratton et al.^34^).

Second, the analyses were repeated for different amounts of data ranging from 1 to 4 runs of data per acquisition type. We found that the similarity between task-regressed and resting-state FIX correlation matrices generalized across all data amounts. The overall correlation strength did change as a function of the amount of data, ranging from ∼0.49 when 1 run of data was used (6 min 50 s) to ∼0.73 when 4 runs of data were used (27 min 20 s) (see Figure S1D for an expanded analysis). Thus, as reported previously, increasing the amount of data improves the stability of functional connectivity estimates.^3,12,38–40^

Third, the similarity of the correlation matrices within the same acquisition type was nearly the same regardless of the acquisition task. For the case of 4 runs of data per dataset, the similarity estimates were 0.79 (FIX1 vs. FIX2), 0.80 (MOT1 vs. MOT2), and 0.81 (EPRJ1 vs. EPRJ2). Thus, no single task acquisition type had an overall benefit over another. This pattern was the same regardless of the amount of data analyzed.

Finally, the similarity of the correlation matrices estimated within individuals was vastly greater than the similarity estimated between individuals (see also Figure S1B). The similarity improved with increasing amounts of data, but the between-individuals correlations for the largest amounts of data (range: 0.30–0.35) were still far lower than the within-individual correlations for the smallest amounts of data (range: 0.45–0.55). This result suggests that high correlations are not a product of the analysis pipelines and emphasizes that within-individual analyses preserve a great deal of correlation structure that is lost when participants are compared with one another or averaged via anatomical registration (see also Gratton et al.,^34^ Finn et al.,^41^ and Gordon and Nelson^42^).

### Networks can be estimated robustly within individuals using task-regressed data

The next results explored whether task data are sufficient to generate precision maps of networks that are practical equivalents to traditional maps generated from resting-state FIX data. This is a particularly important question because, while the results above found a high correspondence between the matrices derived from task-regressed data and traditional resting-state FIX data, they also revealed clear differences. It is thus an open question whether the similarities between different acquisition conditions are sufficient to derive convergent network estimates or whether network estimates change in substantive ways that might impact experiments exclusively using task data.

**Figure 1.**
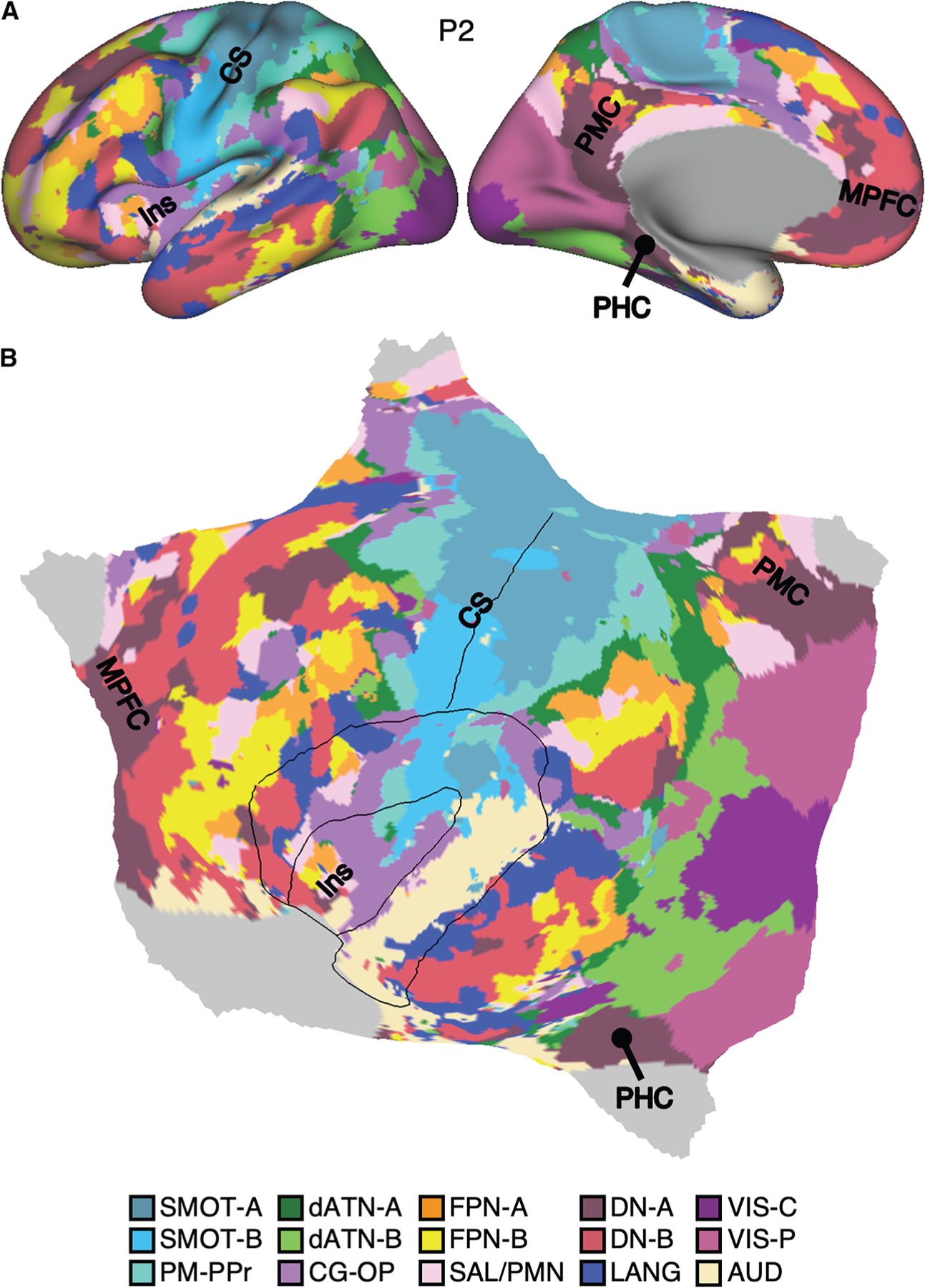
Precision mapping of networks within an individual participant exclusively using task data Inflated (A) and flattened (B) surfaces of the left cerebral cortex display the 15-network MS-HBM estimates for participant P2 using task-regressed data. Distinct networks are represented by different colors, with the network labels provided at the bottom. Somatomotor and visual networks possess primarily local organization, whereas other networks possess widely distributed organization across prefrontal, parietal, and temporal association zones. Reference lines illustrate the inner and outer boundaries of the insula (Ins) as well as along the central sulcus (CS). Additional landmarks are the posteromedial cortex (PMC), parahippocampal cortex (PHC), and medial PFC (MPFC). The network labels are used similarly throughout the figures. SMOT-A, somatomotor-A; SMOT-B, somatomotor-B; PM-PPr, premotor-posterior parietal rostral; CG-OP, cingulo-opercular; SAL/PMN, salience/parietal memory network; dATN-A, dorsal attention-A; dATN-B, dorsal attention-B; FPN-A, frontoparietal network-A; FPN-B, frontoparietal network-B; DN-A, default network-A; DN-B, default network-B; LANG, language; VIS-C, visual central; VIS-P, visual peripheral; AUD, auditory.

Figure 1 provides the answer. When task-regressed data were used exclusively to derive a 15-network multi-session hierarchical Bayesian model (MS-HBM) estimate, the results were remarkably similar to those generated from traditional resting-state FIX data (e.g., Du et al.^19^). The full parcellation of networks for one representative participant, P2, is displayed in Figure 1. The 15 estimated networks are labeled as follows: somatomotor-A (SMOT-A), somatomotor-B (SMOT-B), premotor-posterior parietal rostral (PM-PPr), cingulo-opercular (CG-OP), salience/parietal memory network (SAL/PMN), dorsal attention-A (dATN-A), dorsal attention-B (dATN-B), frontoparietal network-A (FPN-A), frontoparietal network-B (FPN-B), default network-A (DN-A), default network-B (DN-B), language (LANG), visual central (VIS-C), visual peripheral (VIS-P), and auditory (AUD).

**Figure 2.**
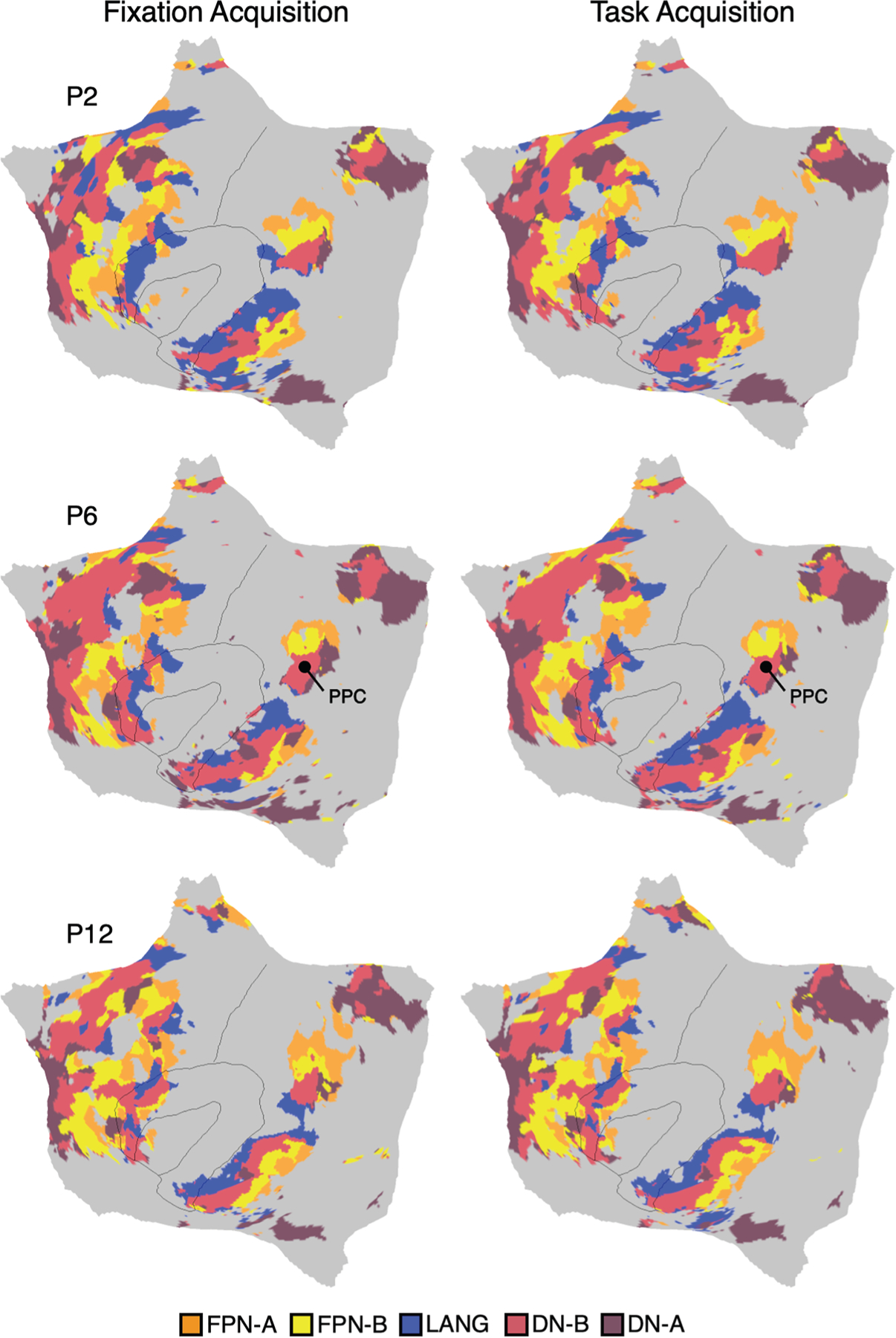
Network estimates are similar between resting-state FIX and task data acquisitions Higher-order association networks from the 15-network MS-HBM estimates are displayed for three representative participants (P2, P6, and P12) for data acquired using traditional resting-state FIX data (left, FIX acquisition) vs. exclusively using task-regressed data (right, task acquisition). The data yielding the network estimate in the left columns is fully independent of the data used in the right columns. The parallel interdigitated distributed networks, FPN-A, FPN-B, LANG, DN-B, and DN-A, are displayed, allowing for direct comparisons of both the broad distributed patterns and idiosyncratic local details. The estimated networks are strikingly similar across both datasets, capturing even idiosyncratic and small regions consistently. A notable example is the juxtaposed spatial segregation between the five networks within the posterior parietal cortex (PPC; labeled for P6). These findings suggest that task-regressed data can effectively estimate brain networks. Similar estimates for all participants are included in the supplemental information. FPN-A, frontoparietal network-A; FPN-B, frontoparietal network-B; DN-A, default network-A; DN-B, default network-B; LANG, language. See also Figures S1 and S2 and Tables S1 and S3.

Figure 2 illustrates a direct comparison of higher-order association networks estimated from FIX acquisition data vs. task acquisition data for three representative participants (P2, P6, and P12). This analysis used all available task data (Table S1) and asked the question of whether, independent of the original parcellations estimated in Du et al.^19^ from FIX acquisition data, the same parcellations would be obtained in practice if only the task data were used. Precision network maps for all participants are available in Figures S2D–S2G. Qualitatively, the network estimates from the two data sources are nearly the same. The idiosyncratic spatial details vary between individuals but were largely preserved within each individual across datasets under independent acquisition conditions but not observed in the group prior (see Figures S2A and S2B). Thus, individuals possess spatial features that are not in the group prior but are identified in task acquisition data with comparable fidelity to traditional approaches.

Quantitative analyses confirmed a high level of correspondence. Across the cortical surface, 79.9% of cortical vertices were assigned to the same network across the two independent resting-state FIX and task-regressed datasets within the same individual. The network-specific overlap percentages were 79.5% (70.5%–86.7%) for FPN-A, 80.5% (76.2%–86.5%) for FPN-B, 82.5% (78.4%–85.9%) for DN-A, 81.4% (75.1%–87.5%) for DN-B, and 77.1% (67.2%–84.5%) for LANG. Overlap percentages for all other networks are reported in Table S3. To place these values in context, which on average are ∼80%, we performed a parallel analysis for two participants from 30 and 31 sessions of MRI data acquisition under uniform resting-state FIX acquisition. The overlap percentages under such intensive and ideal conditions were 86.4% (81.1%–87.4% for FPN-A, 82.8%–90.4% for FPN-B, 88.4%–88.9% for DN-A, 87.7%–91.0% for DN-B, and 77.3%–90.4% for LANG).

Thus, resting-state FIX data and task-regressed data capture a convergent functional architecture of the brain. These findings indicate that task data can be used in practice to estimate precision brain networks that closely align with those estimated from resting-state FIX data.

### Model-free seed-region-based correlations confirm network estimates

The network estimates in Figures 1 and 2 were derived from a 15-network MS-HBM model. This model assumes a specific number of networks and employs a group prior, which might constrain the network estimates to appear similar between acquisition methods. To explore the network estimates in an unbiased manner and examine how well the model captures underlying within-individual correlation patterns of task-regressed data, we conducted an additional set of analyses using a model-free seed-region-based correlation approach. Specifically, we asked whether the correlation patterns from closely juxtaposed seed regions using only the task-regressed data (task acquisition) would align with the network borders estimated from the independent resting-state FIX data (FIX acquisition). This is a strict test of correspondence because it compares independent datasets, each processed with distinct methods that have different assumptions. We focused our analyses on the two adjacent and intertwined networks, DN-A and DN-B.^13,18^ Figures S3E–S3H show results for other networks.

**Figure 3.**
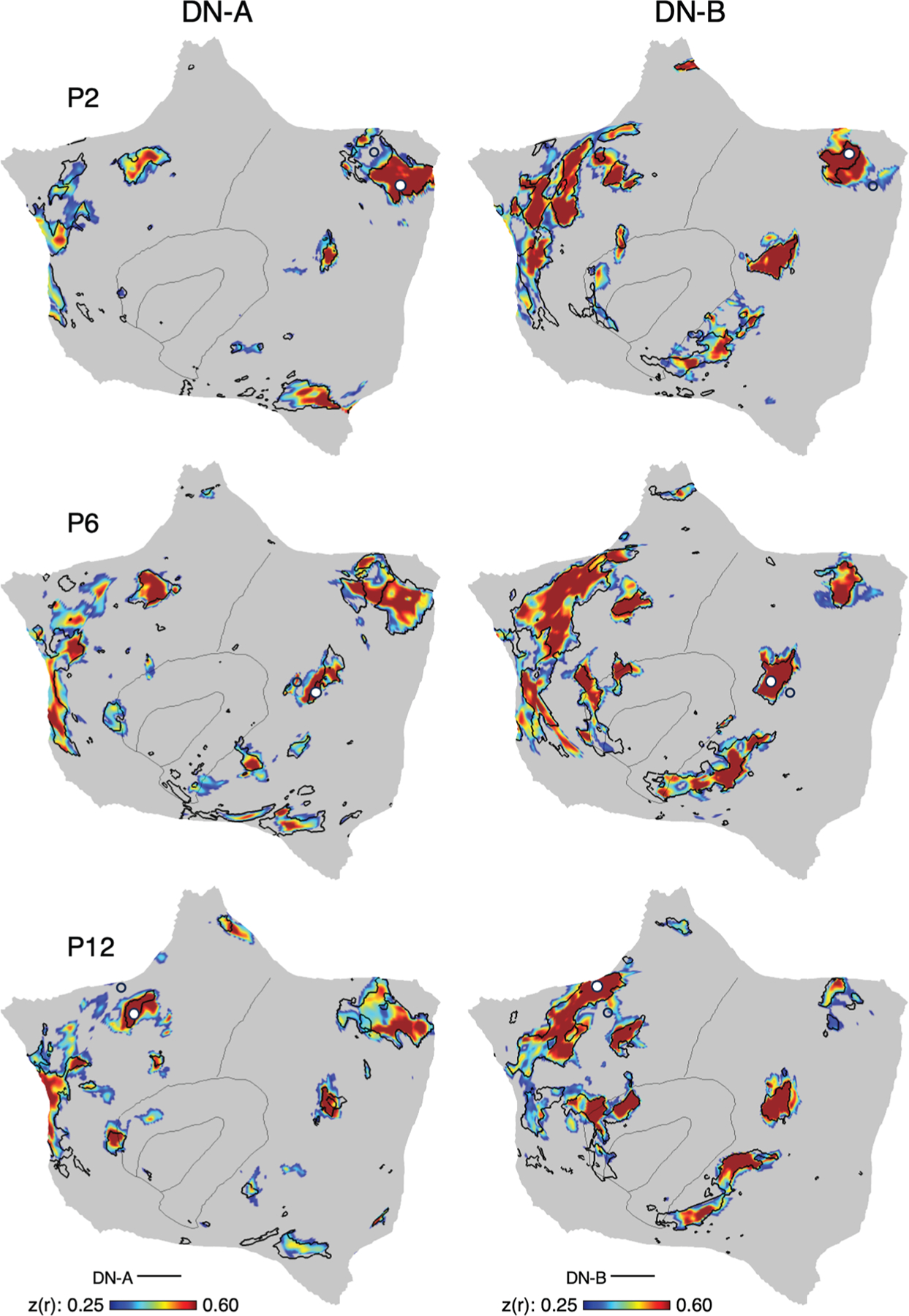
Model-free seed-region correlation maps confirm spatial correspondence between resting-state FIX and task data acquisitions The correlation maps from individual seed regions placed within networks DN-A and DN-B are displayed for three representative participants (P2, P6, and P12). These correlation maps, based exclusively on task-regressed functional connectivity, are plotted as *z*(*r*) with the color scale at the bottom. Black outlines show the boundaries of individual-specific networks estimated from the independent resting-state FIX data within the same individual. White-filled circles mark the seed-region locations, and non-filled circles mark the adjacent seed region within the other (adjacent) network. The positions of the seed regions are moved across association zones between participants to demonstrate that the full distributed extent of DN-A and DN-B can be effectively generated from many component regions. The network borders from resting-state FIX data align well with the spatial correlation properties of the task-regressed data, establishing excellent correspondence between resting-state FIX and task-regressed data. Similar estimates for all participants are included in the supplemental information. DN-A, default network-A; DN-B, default network-B. See also Figures S3 and S4 and Table S1.

Results were robust and support that the spatial correlation patterns align between the acquisition types (Figure 3). Seed regions placed within the network borders defined from the resting-state FIX data yielded substantially overlapping distributed correlation patterns that align with the independent task-regressed data. In P2, the seed regions were placed in posterior midline zones of the DN-A and DN-B networks (Figure 3, top row). Resulting correlation patterns yielded distinct but closely juxtaposed patterns throughout the cortex that aligned well with the independent estimates of the network borders. In P6, analysis of the posterior parietal cortex found similar results (Figure 3, middle row). And in P12, parallel analysis of the dorsolateral prefrontal cortex yielded convergent results (Figure 3, bottom row). Robust correlations remain evident under several conditions: (1) when multiple small seed regions were placed in variable locations within the same general brain zone (Figures S4C and S4E), (2) when the entire brain zone was used as a seed region (Figure S4F), and (3) when multiple widely distributed seed regions across the cortex were used (Figures S4B and S4D). That is, the same result was found across many variations of the analysis. Notably, the observed correlation patterns did not align well with network borders defined from the group prior used in the MS-HBM model (see Figure S2C), again indicating that idiosyncratic features are consistently captured in both within-individual task-regressed and resting-state FIX data but are attenuated in group central tendencies. Comprehensive seed-region-based correlations for all participants are available for representative networks in Figures S3A–S3D. Additionally, to provide a complete visualization of both negative and positive correlations, unthresholded correlation maps for the seed regions shown in Figure 3 are available in Figure S4A.

### Network estimates exclusively from task-regressed data predict functional specificity

Our next result illustrates the practical implications for precision brain mapping under tightly controlled conditions (e.g., matched amounts of FIX and task acquisition data; Table S2). Construct validity was tested by exploring whether task-regressed data yield network estimates that can serve as localizers for prospective explorations. That is, if one collected exclusively task data and estimated networks as a form of within-individual localizer, would the results be similar to traditional approaches? We focused specifically on the functional specialization of three association networks—DN-A, DN-B, and LANG—that are spatially juxtaposed throughout the distributed association zones but established to be functionally distinct.

**Figure 4.**
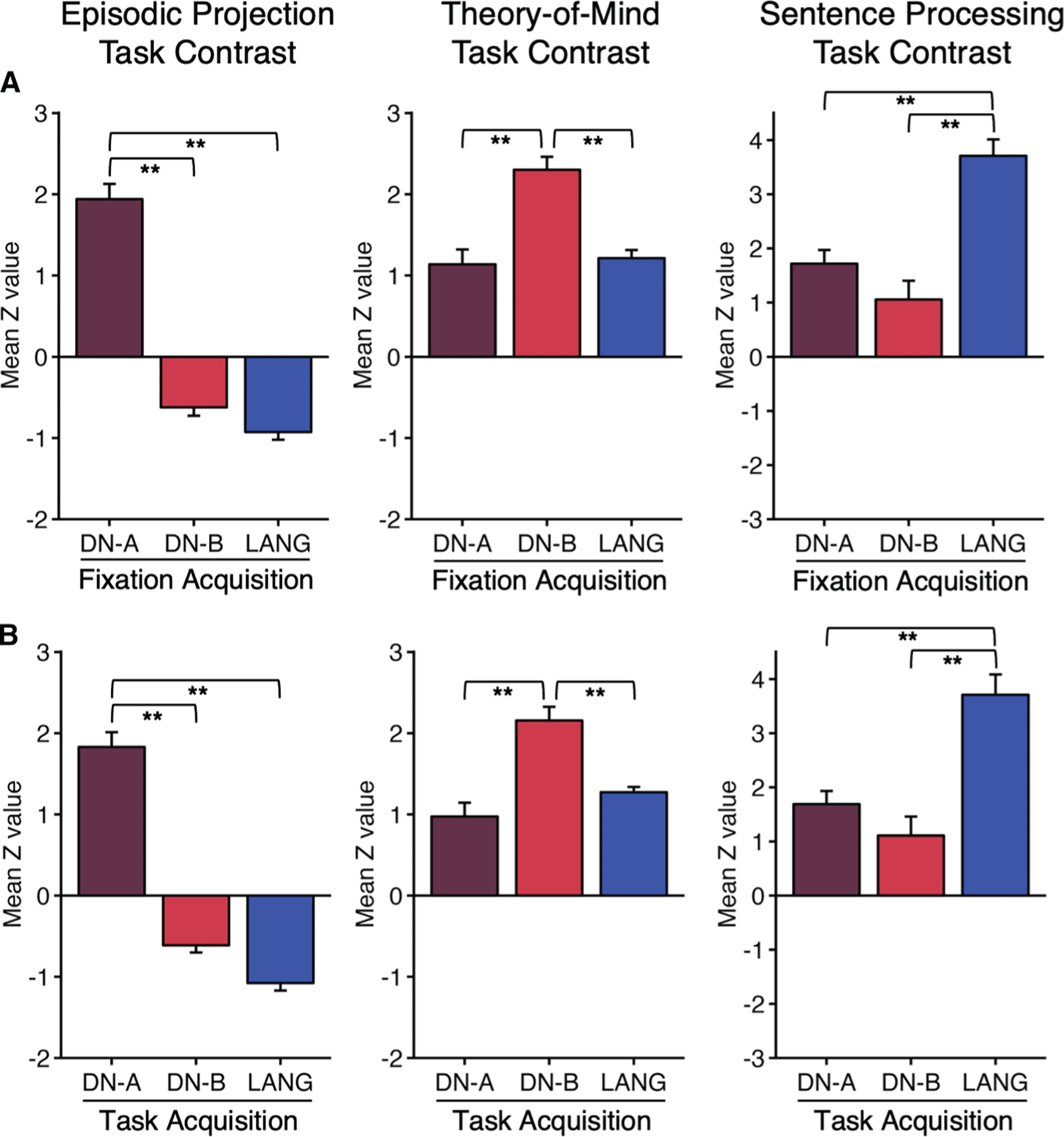
Networks estimated exclusively using task data predict independent task responses as well as traditional resting-state FIX network estimates As a test of validity, network estimates from traditional resting-state FIX runs (top, FIX acquisition) were contrasted with network estimates exclusively using task runs (bottom, task acquisition) in their ability to prospectively predict functional dissociations in independent task data. (Top) Bar graphs quantify the responses of EPRJ (left), ToM (middle), and SENT task (right) contrasts as mean *z* values (*N* = 11) across the multiple *a priori*-defined networks using traditional resting-state FIX data. Note the clear and robust triple dissociation (see Du et al.^19^). (Bottom) Similar bar graphs quantify the responses across the multiple *a priori*-defined networks derived exclusively from task-regressed data. The task data used to define the networks was fully independent of the tasks and constructs tested in the bar plots. Each plot displays data from a distinct task contrast; each bar represents a distinct network. The full 3 × 3 interaction (network by task contrast) is significant (*p* < 0.001) for both acquisition types. All pairwise comparisons are also significant for both acquisition types, confirming the full triple dissociation. Asterisks indicate a value is significant (**p < 0.001). See also Figure S4 and Table S2.

Figure 4 displays the functional response levels for these three networks when the networks were defined by resting-state FIX data (FIX acquisition) vs. exclusively task-regressed data (task acquisition). Note that the functional responses plotted are from data that are independent of the data used to define the networks in both cases. DN-A is robustly and preferentially activated for the EPRJ task contrast, DN-B for the theory-of-mind (ToM) task contrast, and LANG for the sentence processing (SENT) task contrast. Formal statistical analysis using repeated measures ANOVA on network-level task response revealed a significant 3 × 3 interaction between the effects of task contrast and network derived from independent task-regressed data (F(4, 40) = 54.0, *p* < 0.001) and the resting-state FIX data (F(4, 40) = 73.3, *p* < 0.001). For reference, we also conducted a parallel functional response analysis using the prior group from the MS-HBM model (see STAR Methods). As shown in Figure S4G, functional responses increase when moving from the group prior to within-individual network estimates, again revealing the benefit of conducting analyses within the idiosyncratic anatomy of the individual: EPRJ responses increased by 56.0%, ToM responses increased by 29.6%, and SENT responses increased by 51.7%. The gain was even more pronounced when the analysis was conducted within anatomically complex zones of cortex where anatomical juxtapositions are most variable between people, including lateral prefrontal zones (EPRJ increased by 119.5%; ToM responses increased by 62.3%; and SENT responses increased by 67.5%), medial prefrontal zones (EPRJ increased by 130.8%; ToM responses increased by 24.7%; and SENT responses increased by 207.4%), and temporal association zones (EPRJ increased by 276.3%; ToM responses increased by 20.3%; and SENT responses increased by 45.7%).

These results indicate that network estimates from task-regressed data predict functional response properties in independent contrasts similar to parallel analyses using traditional resting-state FIX data.

### Pooling task-regressed and resting-state FIX data stabilizes functional correlation matrices

A potential use of task-regressed data is to pool it with resting-state FIX data from existing or prospective data acquisitions to increase power (e.g., see Elliott et al.,^30^ Kraus et al.,^32^ Gratton et al.,^34^ and Satterthwaite et al.^43^). If pooling data across acquisition types can increase statistical power to estimate networks, it opens multiple opportunities for novel scientific discovery. To quantitatively illustrate the potential gain from data pooling, we assessed the similarity of functional correlation matrices using data from a single resting-state FIX run, a single MOT task run (after regression of the task structure), and their combination within the same individuals. Similarity was measured as the correlation between functional connectivity matrices from independent test and retest datasets within each condition (1 FIX vs. 1 FIX; 1 MOT vs. 1 MOT; 1 FIX + 1 MOT vs. 1 FIX + 1 MOT).

**Figure 5.**
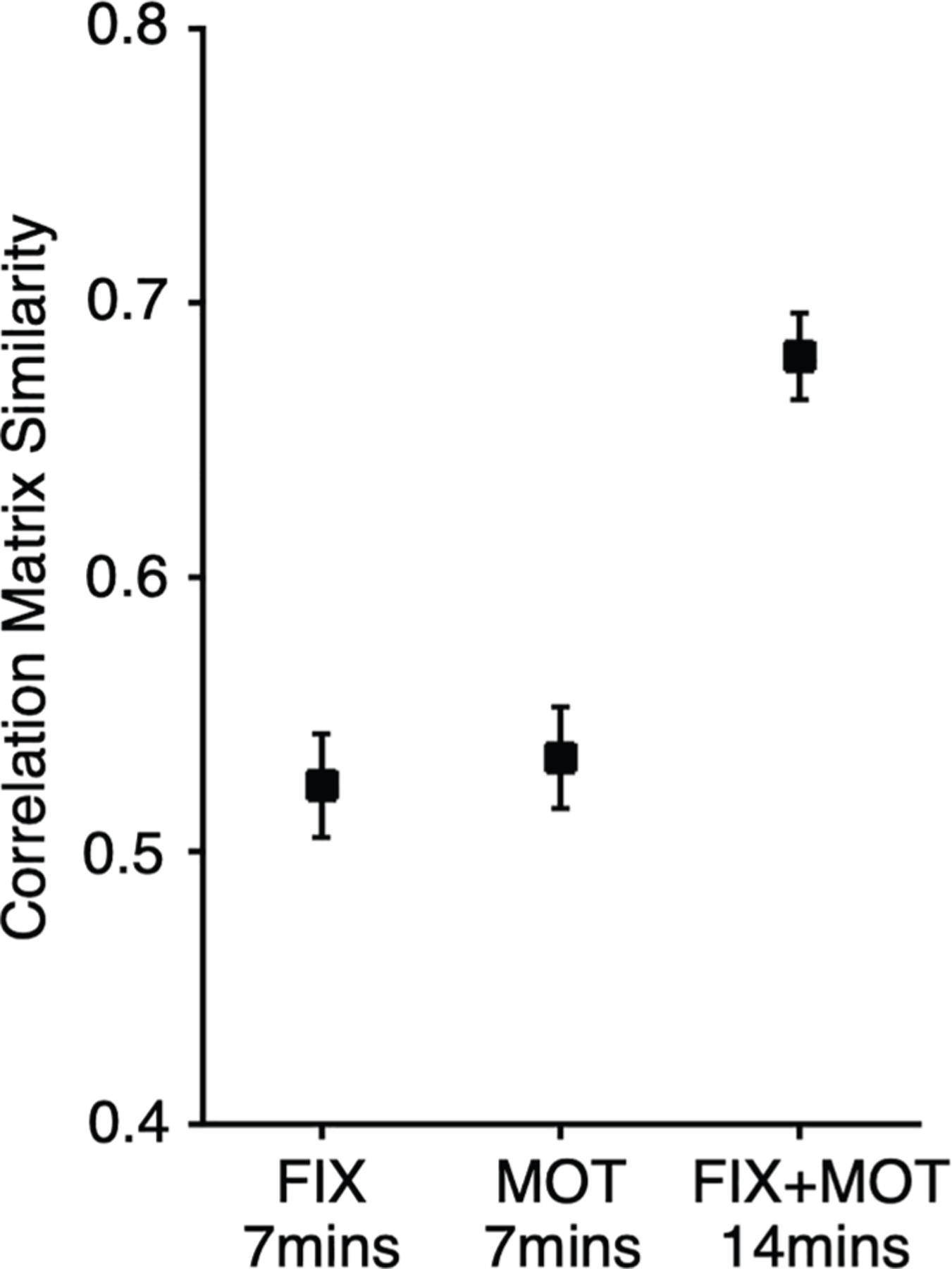
Stability of within-individual functional correlation matrices is enhanced by pooling task and resting-state FIX data The mean similarity values between functional correlation matrices using a single resting-state FIX run, a single MOT task run, and a combination of both are plotted. Similarity was quantified as the correlation between independent test and retest functional connectivity matrices for each condition, calculated within each participant and then averaged (*N* = 11). Error bars represent the standard error across participants. The similarity was improved from 0.52 to 0.53 with one run of either data type to 0.68 when data were pooled. Pooling resting-state FIX and task-regressed data enhances the stability of functional correlation matrices due to the increased amount of available data.

Mean similarity values between functional connectivity matrices were 0.52 with 1 FIX run and 0.53 for 1 MOT alone (Figure 5). Notably, when pooling FIX and MOT runs, similarity significantly increased to 0.68 (a significant gain over either the MOT [*t* = 6.02, *p* < 0.001] or FIX [*t* = 6.34, *p* < 0.001] runs in isolation). These results indicate that the stability of within-individual functional connectivity matrices is improved by combining resting-state FIX data with task-regressed data, as predicted by the multiple findings that suggest task-regressed data are practical equivalents to traditional resting-state FIX data. To directly illustrate the utility of this benefit, in the next section, we mapped the detailed organization of thalamic association zones within individuals by pooling extensive existing resting-state FIX and task data.

### Pooled data reveal juxtaposed thalamic subregions linked to distinct cortical association networks

The thalamus is a small structure tightly packed with adjacent individual nuclei, each with its own complex spatial topography. To demonstrate a newly enabled application of task-regressed data, we mapped the detailed organization of the thalamic association zones by pooling extensive within-individual resting-state and task-regressed data, tripling the amount of data available for analysis. We found segregated representations of each of the five cortical higher-order association networks in most individuals (FPN-A, FPN-B, LANG, DN-B, and DN-A).

**Figure 6.**
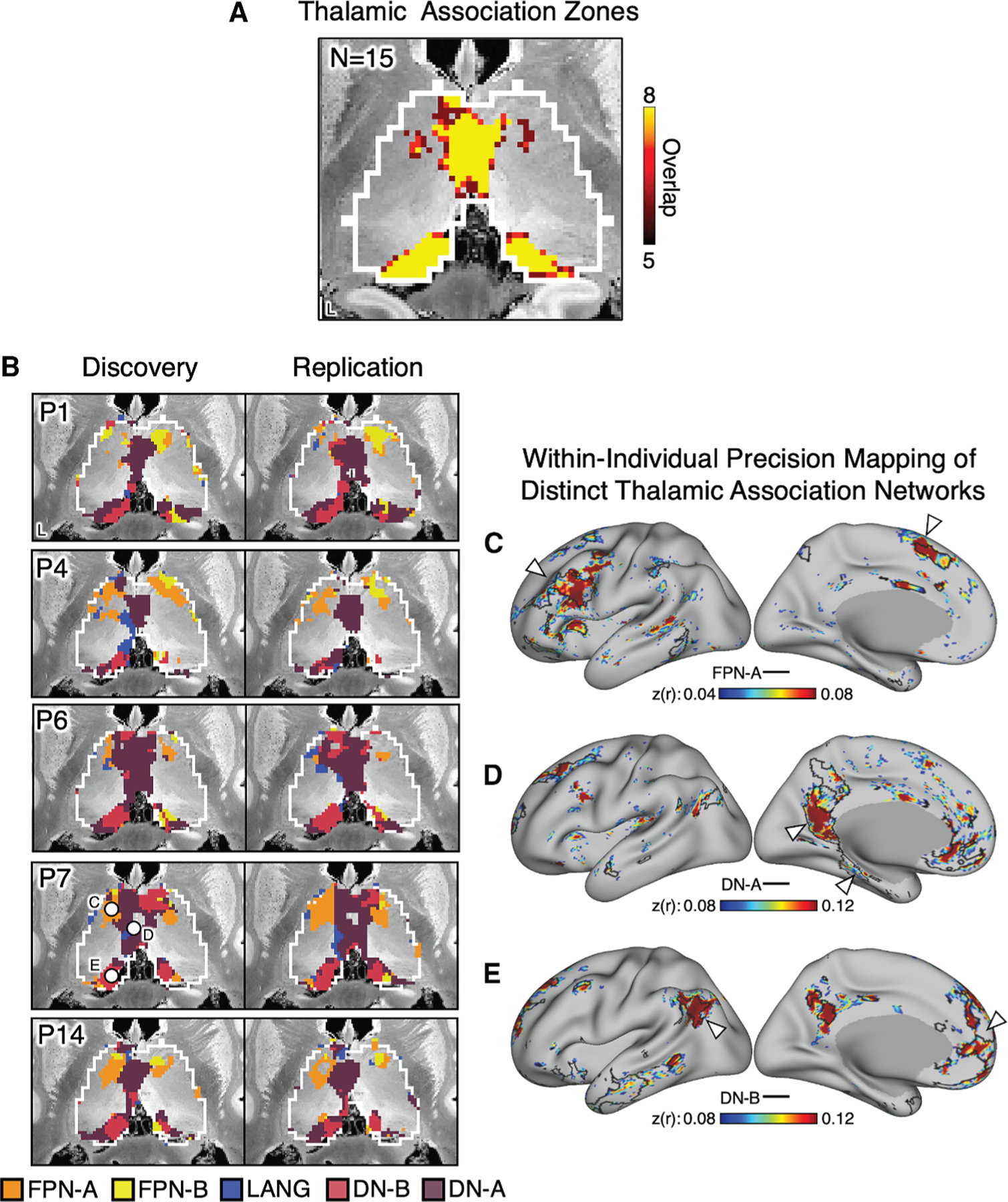
The idiosyncratic organization of thalamic association zones within individuals is evident when resting-state FIX and task data are combined The organization of thalamic association zones was examined by pooling *all* available resting-state FIX and task data to dramatically boost signal within individuals. (A) A transverse section reveals the most consistent thalamic voxels assigned to any higher-order association network (FPN-A, FPN-B, LANG, DN-B, or DN-A). This consensus map is shown overlaid on the high-resolution thalamic atlas.^44^ The higher-order association zones generally fall along the midline thalamic nuclei. The color scale reveals the number of participants (out of 15) that had any higher-order association network assigned to that voxel of the thalamus. (B) Thalamic organization is visualized for independent discovery and replication datasets within individuals (P1, P4, P6, P7, and P14) to reveal the estimated locations of the five distinct association network representations. Note that the idiosyncratic anatomical details differ between individuals but are reliable within a person. Further note that the distinct association zones have a general topographic pattern with FPN-A and FPN-B positioned anteriorly, DN-A falling along an extended midline zone, and DN-B positioned along the posterior midline. In many cases though, multiple disjointed anterior and posterior subregions are linked to the same network. Data from all participants are available in the supplemental information. (C–E) Cortical functional connectivity patterns are shown on the inflated surface for three distinct thalamic seed regions (from P7 as displayed by white circles in B). White arrows highlight hallmark features that distinguish the networks as described in the text. L, left; FPN-A, frontoparietal network-A; FPN-B, frontoparietal network-B; DN-A, default network-A; DN-B, default network-B; LANG, language. See also Figures S6–S8.

We first mapped the locations of the collective set of higher-order association networks to the thalamus in all participants, yielding a consensus estimate of where, in aggregate, higher-order association networks are represented in the thalamus. Figure 6A illustrates the results. Consistent with the literature, including studies using group averaging, higher-order association networks were linked to the thalamic midline extending from the anterior nucleus through the mediodorsal nucleus to the medial pulvinar (Figure 6A; see Figure S5A for an expanded presentation).

We next mapped the five networks separately within each individual participant. While the idiosyncratic anatomical details differed between people, all five networks were identified and found to be reliable across datasets in most individuals. Figure 6B illustrates results from independent discovery and replication datasets in representative participants (results from all individuals can be found in Figure S5C). The relative spatial positions of the thalamic subregions roughly recapitulated their cortical organization with the same general organization in each individual: subregions corresponding to FPN-A and FPN-B were adjacent more anteriorly, next to subregions associated with the LANG, DN-B, and DN-A networks. Moreover, the thalamic subregions linked to the LANG network preferentially exhibited left lateralization consistent with the network’s representation in the cortex.

A final test of segregation of the thalamic association subregions was undertaken by examining whether closely juxtaposed seed regions placed within the thalamus could recapitulate the spatially distinct cortical networks. For this analysis, seed regions were individually placed within the five different network representations in each individual’s thalamus. Figures 6C–6E illustrate the cerebral correlation patterns associated with three thalamic seed regions in a representative participant, P7. The seed regions placed in the small FPN-A thalamic subregion produced a correlation map that largely aligned with the boundaries of the cortical FPN-A network, including its canonical dorsolateral prefrontal cortex and anterior cingulate regions (Figure 6C). Similar specificity was observed for seed regions placed in the DN-A (Figure 6D) and DN-B (Figure 6E) thalamic subregions, with the seed region in DN-A yielding canonical parahippocampal and retrosplenial cortex regions and the seed region in DN-B yielding regions along the frontal midline, temporoparietal junction, and the central portion of the precuneus (see Braga et al.^45^ for reference). Additional results from this participant are provided in Figure S7B (see Figures S6–S8 for similar analyses from all participants). Specificity was found across many of the participants, with some participants yielding clearer segregation between networks than others, but with all networks revealing localization to segregated thalamic subregions across multiple independent participants.

### Task data can be used to simultaneously estimate network organization and extract task responses

A critical implication of the present findings is that studies can exclusively use task data to estimate network topography within an individual’s own anatomy (from the task-regressed component of the data) while simultaneously extracting the task response from the localized networks. To illustrate such dual-purpose analysis and test whether it can yield unbiased estimates of the task response, we localized networks from both traditional resting-state FIX data as well as task data in the same set of participants (see Table S2). Then, the task response was extracted from within-individual networks defined either exclusively from resting-state FIX data or alternatively from the task-regressed data. The ODDBALL task elicits a robust, transient response in the SAL and CG-OP networks when visual oddballs are detected and suppresses responses in DN-A and DN-B.^19^

**Figure 7.**
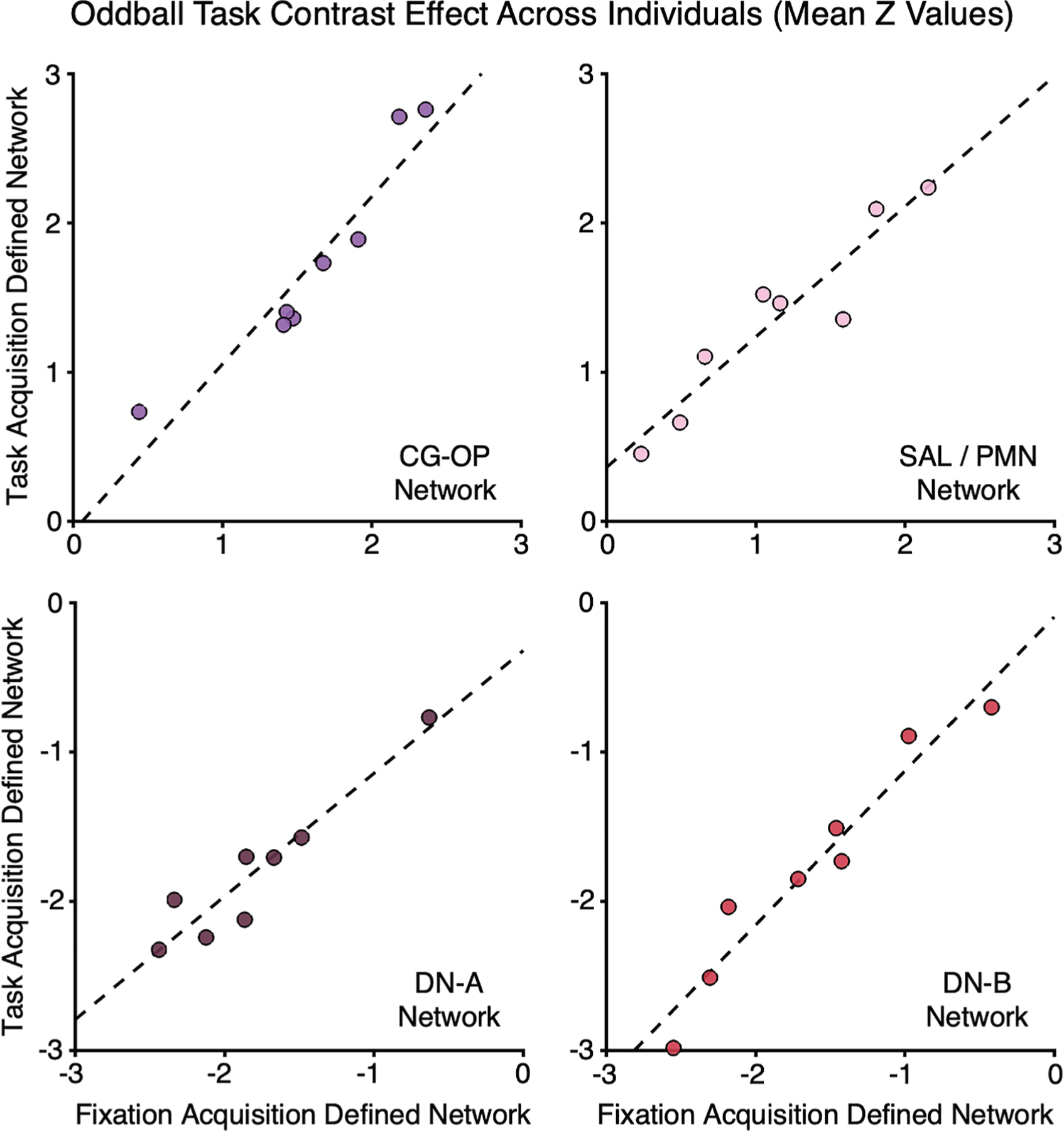
Between-individual differences in task response can be obtained from task-regressed network estimates without acquiring separate resting-state FIX data Data from 8 participants, each with five ODDBALL task runs and five resting-state FIX runs, were independently input into the 15-network MS-HBM model to estimate within-individual precision networks. ODDBALL task responses were then quantified within CG-OP (A), SAL/PMN (B), DN-A (C), and DN-B (D), using precision networks defined from the resting-state FIX and task acquisitions separately. Each dot represents an individual participant, and dot colors correspond to network color in Figure 1. Across four networks, strong correlations were observed between functional response levels in networks defined from resting-state FIX data and task-regressed data, indicating the task data can be used to estimate networks within an individual’s idiosyncratic anatomy. This finding suggests a novel and efficient strategy of using only task-based data to estimate networks within an individual’s own anatomy as well as estimate the task response within those individualized networks.

A strong positive response of the ODDBALL effect was observed in both the CG-OP and SAL/PMN networks, while task-induced deactivation was robust in both the DN-A and DN-B networks, consistent with previously reported effects.^19^ The response levels varied considerably between individuals (Figure 7). Crucially, the functional response levels extracted from networks defined from resting-state FIX data were highly correlated with levels from networks exclusively estimated from the task-regressed data (r = 0.94 for CG-OP; r = 0.94 for SAL/PMN; r = 0.94 for DN-A and r = 0.97 for DN-B). The variability did not appear to be attenuated or biased, as the best-fit line was near to the identity line (see Figure 7). Thus, the same task data can serve a dual role, enabling both precision network estimates and traditional task response analysis. This approach offers a practical means to reduce MRI session time, associated costs, and participant burden, while retaining sensitivity to individual differences in network estimates and task response level.

**Figure 8.**
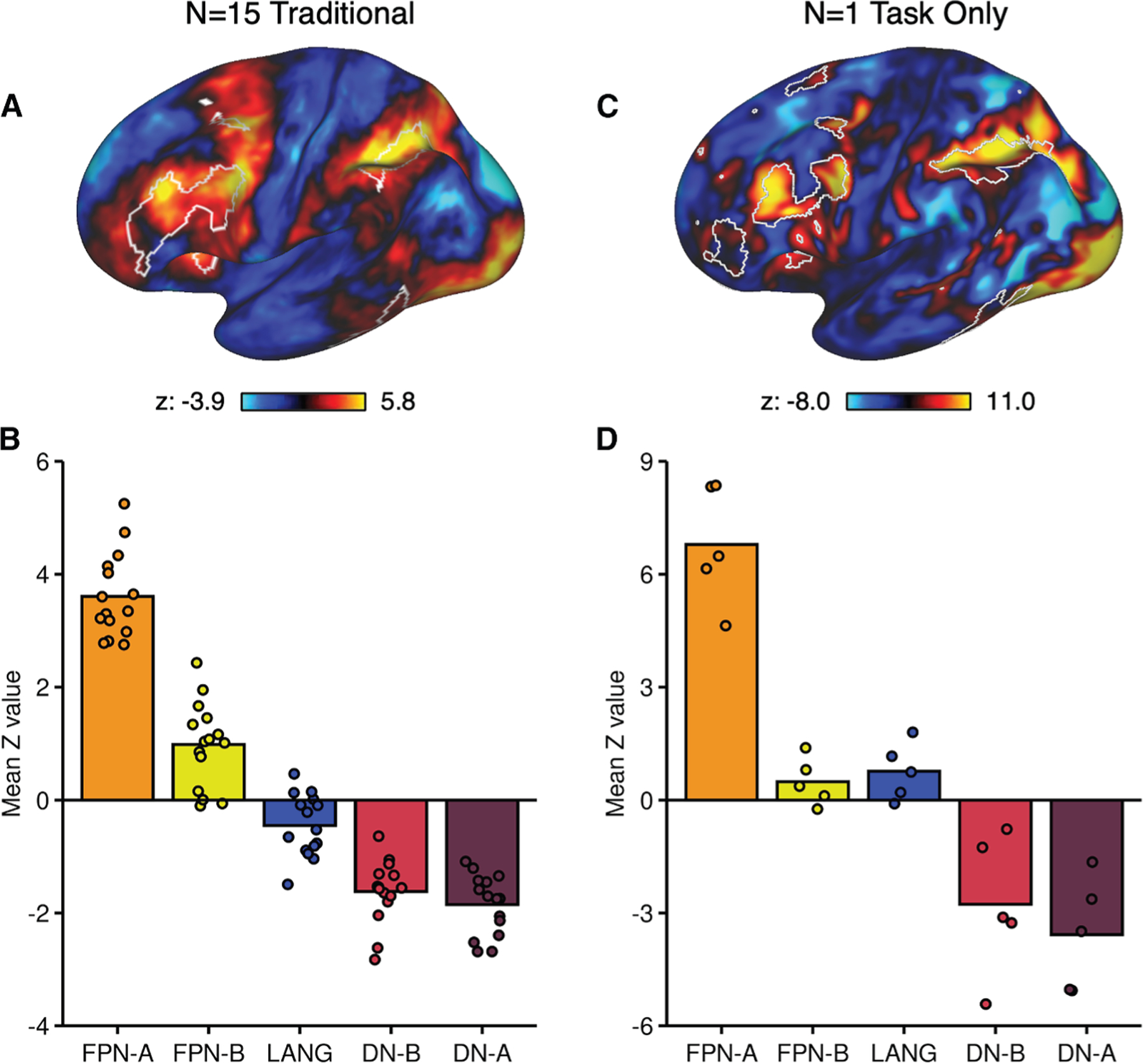
Networks and working memory task response extracted from a single (∼1 h) MRI session An example of network estimation and task response extraction from a participant using only task data. (A) The mean task response for blocks of the NBACK working memory task as contrasted to FIX across 15 individuals from Du et al.^19^ show regions of response increase (red) and decrease (blue). Note the robust response in frontal and parietal regions. The white outline demarcates the estimated group-level FPN-A network (DU15NET-consensus; see Du et al.^19^). (B) Mean response is plotted separately for each higher-order association network (FPN-A, FPN-B, LANG, DN-B, or DN-A). Each point represents the mean response from an individual participant. Note the robust response in FPN-A. (C) The mean task response for a single session of data from a new individual showing an individualized, robust response to blocks of the NBACK working memory task as contrasted to FIX. The white outline demarcates the individually estimated FPN-A network from the task-regressed component of the data. (D) Mean response is plotted separately in this individual for each higher-order association network (FPN-A, FPN-B, LANG, DN-B, or DN-A). The within-individual precision networks were estimated from the task-regressed data using the 15-network MS-HBM model, and then the responses were extracted from each network. Each point represents the response from one of the 5 runs of NBACK data acquired using letter stimuli (matching Du et al.^19^). Note the robust response in FPN-A. The surfaces in this figure are rotated along the y plane to better show the intraparietal sulcus. FPN-A, frontoparietal network-A; FPN-B, frontoparietal network-B; DN-A, default network-A; DN-B, default network-B; LANG, language.

To prospectively demonstrate efficient network estimation and task response extraction, we obtained precision networks in a new participant using only NBACK task data from a single session and then extracted the task response for each run from the same data session. Plotted in Figure 8 are the original results from Du et al.^19^ compared with the new results obtained from the single-task data session. Among the higher-order association networks, there was a strong preferential response in FPN-A as compared with spatially distinct (but nearby) association networks (Figure 8). This result reveals, as a proof-of-concept, that a single MRI session of ∼1 h in length can be used both to estimate precision brain networks and quantify meaningful task responses.

## DISCUSSION

We provide a robust procedure for precision mapping of brain networks within individuals using only task data. Building on prior findings,^23,25–27,29–32,34^ we found that networks estimated from task-regressed data are highly similar to those derived from traditional resting-state FIX acquisitions (Figures 1, 2, and 3), and further that they predict independent functional dissociations (Figure 4). These findings lay a foundation for novel experimental designs and discovery opportunities. In one application of these methods, signal quality within individual participants was improved by pooling extensive task and resting-state FIX data, enabling the identification and replication of segregated thalamic association regions (Figure 6). In another example, we demonstrated that the same task data can be used to map individualized networks while simultaneously extracting task-related responses (Figures 7 and 8). Overall, our findings suggest that task data alone are sufficient to generate precise network estimates within individuals, supporting a wide range of potential applications.

### Within-individual precision mapping of brain networks exclusively using task data

Functional neuroimaging with MRI has commonly used two broad classes of methods—methods that employ task-based contrasts to characterize regions responding to structured task demands and methods that analyze spontaneous fluctuations to detect the intrinsic architecture of brain networks (see Finn^46^ for a historical perspective). Both methods have contributed to important discoveries. However, there has been a practical tension between the two types of methods because they are acquired across different scans. Given limitations on cost and participant burden, imaging sessions typically prioritize one type of data acquisition or strike an uneasy balance. Past data acquired for the purpose of measuring task responses has rarely been used for functional connectivity-based network estimation (for early exceptions, see Greicius et al.,^11^ Fair et al.,^23^ Greicius et al.,^47^ and Andrews-Hanna et al.^48^).

For example, in the Human Connectome Project (HCP), data were acquired over 2 days with 4 runs of data dedicated to resting-state FIX scans amenable to analyses using functional connectivity and 14 runs dedicated to task paradigms.^49^ Similarly, the recent UK Biobank effort, which was substantially constrained by scan length limits, included one run of resting-state FIX data and one run of face emotion processing task data.^50^ Broadly across the field, including in our laboratory’s efforts, scan runs acquired for the goal of estimating network organization are separated from runs acquired to measure task response.

Building from the insight that the correlation of intrinsic fluctuations between regions is similar for resting-state FIX acquisitions and task acquisitions (after regression of the task structure; Fair et al.^23^), we developed and tested a procedure to estimate brain networks within individuals exclusively from task data. The results were clear. Network estimates from task data converged with traditional estimates from resting-state FIX data. The estimates were qualitatively similar to the eye (Figure 2), quantitatively similar in terms of estimates of overlap, and predicted preferential responses in external task data (Figure 4). That is, the task-regressed network estimates were highly effective functional localizers.

### Implications for task designs and analysis

The present findings have multiple implications. First, large amounts of task data have been collected through local efforts and large-scale consortia (e.g., Gordon et al.,^14^ Du et al.,^19^ Van Essen et al.,^49^ Miller et al.,^50^ Volkow et al.,^51^ and Allen et al.^52^). These previously acquired data can be reanalyzed with the present approaches to estimate networks using functional connectivity. Second, for already collected datasets that have both task and resting-state acquisitions, power can be increased for estimating networks by combining and using all of the data. Our results (and those across the field broadly) suggest that the amount of data utilized for network estimation is critical. Thus, utilization of all data may allow for sufficient data pooling to stabilize within-individual precision estimates when passively acquired data are limited. Figure 5 illustrates that functional connectivity matrices are stabilized by augmenting the resting-state FIX runs with task-regressed data. Using this approach, we were able to identify segregation of distinct association networks along the thalamic midline (Figure 6). Moreover, these thalamic association subregions recapitulated spatially distinct cerebral cortical networks. These findings support the growing framework that positions the thalamus, basal ganglia, and cerebellum as nodes within an integrated brain-wide circuit involving the cerebral cortex (Bostan and Strick^53^; see also Buckner^54^). Our prior efforts to map the thalamus, from these same participants, that used only the resting-state acquisitions were ambiguous. By combining the task data, we were able to triple the amount of data analyzed within each participant and to enable precise mapping of this small, deep brain region.

As a counterintuitive possibility, in situations where task and resting-state acquisitions are both acquired, the task data, via the methods proposed here, can be used to estimate the topography of networks within individuals, and then the localized networks can be used to compute within- and between-network correlations in the separate resting-state data. This seemingly odd task design, which to our knowledge has never been conducted, would allow independent data to be used to estimate the location of target regions prior to analysis of region-to-region correlations that may fluctuate over time (e.g., Lynch et al.^16^) or in response to an intervention.^55^ Using the task-regressed data will allow the networks to be estimated separately from the data used to measure and track changes in the spontaneous correlations between regions in a spatially unbiased manner.

Finally, we found that task data can be effectively leveraged to estimate precision networks within an individual’s idiosyncratic anatomy, while simultaneously extracting the network-level functional responses (Figure 7). As a proof-of-concept of this application, we measured the strong, preferential response in the FPN (specifically FPN-A) from a single (∼1 h) MRI session within an individual using data exclusively acquired during tasks (Figure 8). These findings suggest that future data collections might consider acquiring only task data and using that data to both estimate networks and also to quantify the evoked task response from regions within those networks. This key opportunity is worth elaborating.

Many recent findings have demonstrated the importance of taking into consideration the idiosyncratic anatomy of the individual in functional analyses (e.g., Laumann et al.,^12^ Braga and Buckner,^13^ Gordon et al.,^14,15^ Fedorenko et al.,^56^ Smith et al.,^57^ Noyce et al.,^58^ DiNicola et al.,^59^ and Somers et al.^60^; for further discussion, see Gratton and Braga^61^ and Laumann et al.^62^). In some studies, one set of task runs is used to localize regions and networks, which are then examined in independent task data to quantify response properties within the localized anatomy of the individual. In other studies, network estimates or regional (areal) parcellations are constructed from resting-state FIX or similar data. These estimates are then used as localizers to quantify response properties in independently collected task runs. The present results suggest that task data can solely be acquired. The task-regressed data can be used to estimate the spatial extent of regions and networks, and then the spatially localized task responses are obtained from the same data via the standard output of the general linear model (GLM) analysis. This novel task design is efficient because all of the runs acquired to estimate the task responses are also used to estimate the networks.

### Limitations of the study

While the present findings indicate that brain networks estimated from task acquisitions are highly similar to those derived from resting-state FIX data, the results do not address the source or meaning of the state-dependent components of the data. Replicating prior analyses, we found that modest between-task differences persisted even after regression of the task paradigm structure. Without regression, the correlation structure differences were even greater (Figure S1C). The differences in correlational structure between tasks may provide insight into how networks interact and how dynamic states emerge that support momentary task demands. Such differences are intentionally minimized by the present methods that extracted the most stable aspects of the correlation structure. Future research should more comprehensively investigate how state- or task-specific factors influence network correlations, perhaps incorporating graph theoretical measures as well as tasks designed to maximally shift cognitive states.

## RESOURCE AVAILABILITY

### Lead contact

Requests for further information and resources should be directed to and will be fulfilled by the lead contact, Randy L. Buckner (randy_buckner@harvard.edu).

### Materials availability

This study did not generate new, unique reagents.

### Data and code availability

Individual participant data associated with this work are available through the NIH repository (https://nda.nih.gov) and Harvard Dataverse (https://doi.org/10.7910/DVN/CEUUNG). Parcellation, SNR, and task contrast maps related to this manuscript are available on Balsa (https://balsa.wustl.edu/study/zK166). Task descriptions, contrast descriptions, and code are provided on Harvard Dataverse (https://doi.org/10.7910/DVN/AVB4BW). The 15-network group prior and related DU15NET atlases are available at FreeSurferWiki (https://freesurfer.net/fswiki/CorticalParcellation_DU15NET). Functional connectivity between brain regions was calculated in MATLAB (version 2019a; http://www.mathworks.com; MathWorks, Natick, MA) using Pearson’s product moment correlations. FreeSurfer v6.0.0, FSL, and AFNI were used during data processing. Model-free seed-region analyses were performed using Connectome Workbench v1.3.2. All statistical analyses were performed using R v3.6.2. Network parcellation and analyses were performed using code available on Github (http://github.com/bucknerlab/PrecisionNetworkMapping) and by Kong et al.^63^ (https://github.com/ThomasYeoLab/CBIG/tree/master/stable_projects/brain_parcellation/Kong2019_MSHBM).

## STAR★METHODS

### Overview

The goal of these analyses was to explore if precision mapping of brain networks can use task data to yield equivalent estimates to those traditionally generated from resting-state fixation data. The analyses proceeded in three phases. First, equivalent amounts of task and resting-state data were used to define correlation matrices that were compared to one another to determine their similarity. The correlation matrices are the basis of functional connectivity analyses so directly contrasting the matrices provides information about correspondence without any assumptions about network models or region definitions. Second, we asked the question of whether task data is sufficient to generate precision maps of networks that are equivalent to traditional maps generated from resting-state fixation data. Correlation matrices from task data were first used to generate within-individual precision maps of networks. The network estimates from the task data were then directly compared to network estimates that used only traditional resting-state fixation data. As a final analysis, construct validity was tested by asking the question of whether task data yield network estimates that could serve as localizers for prospective explorations. Equivalent amounts of data from task acquisitions and resting-state acquisitions were used to define networks. Then, the network estimates were used prospectively to measure functional responses in independent task data that was not used to generate the networks. The critical test was whether known, differential response patterns could be obtained similarly for task-based network estimates as compared to estimates from traditional resting-state acquisitions.

### EXPERIMENTAL MODEL AND STUDY PARTICIPANT DETAILS

#### Participants

The main analyses used fifteen right-handed native English speakers (labeled P1 to P15) who participated for monetary compensation (aged 18 to 34; mean = 22.1 yr, SD = 3.9 yr; 9 female). The data were previously used for traditional resting-state functional connectivity analyses and reported in papers focused on the cerebral cortex,^19^ the cerebellum,^69^ the striatum,^70^ and the hippocampus.^71^ Here the task-based data were reanalyzed with a focus on estimating precision brain networks using task-regressed functional connectivity. Participants for the main cohort were scanned across 8–11 MRI sessions. Each session included multiple resting-state fixation and task-based runs. Four of the fifteen participants were excluded from network estimates based solely on task-regressed data due to insufficient task data, leading to the inclusion of eleven participants (see Table S1; also refer to Table 1 in Du et al.^19^). Usable resting-state runs ranged from 16 (P2) to 24 (P12) runs. Usable task-based runs ranged from 48 (P3) to 70 (P12) runs (see Table S1). For thalamic network analysis, where task-regressed and resting-state fixation data were pooled, 15 participants were included. An additional participant (age 26, female) was scanned during a single MRI session using only tasks to test whether networks and task responses could be extracted simultaneously. Participants with a history of neurological or psychiatric illness were excluded. All participants provided informed consent using protocols approved by the Harvard University Institutional Review Board.

### METHOD DETAILS

#### MRI Data Acquisition

Details of the methods have previously been reported in Du et al.^19^ with relevant portions repeated here. Scanning was performed at the Harvard Center for Brain Science using a 3 T Siemens MAGNETOM Prisma^fit^ MRI scanner and a 32-channel phased-array head-neck coil (Siemens Healthineers AG, Erlangen, Germany). Foam and inflatable padding minimized head movement. Participants viewed a rear-projected display positioned to optimize comfortable viewing. Eyes were video recorded using an Eyelink 1000 Core Plus with Long-Range Mount (SR Research, Ottawa, Ontario, Canada). MRI data quality was monitored during the scan using Framewise Integrated Real-time MRI Monitoring (FIRMM; Dosenbach et al.^72^).

Each participant was scanned across 8–11 sessions most often over 6 to 10 wks. A few participants had longer gaps between the first and last MRI sessions up to one year. Each session involved multiple fMRI runs to be used for functional connectivity analysis, for a total of 16 to 24 resting-state fixation runs, and 48 to 70 task-based runs obtained for each individual. Blood oxygenation level-dependent (BOLD) acquisition parameters were: voxel size = 2.4 mm, TR = 1,000 ms, TE = 33.0 ms, flip-angle = 64°, matrix 92 × 92 × 65 (FOV = 221 × 221), 65 slices covering the full cerebrum and cerebellum. Each resting-state fixation run lasted 7 min 2 sec (422 frames with the first 12 frames removed for T1 equilibration). Dual-gradient-echo B0 fieldmaps were acquired.

High-resolution T1w and T2w structural images were acquired based on the Human Connectome Project (HCP) sequences.^73^ T1w magnetization-prepared rapid gradient echo (MPRAGE) parameters: voxel size = 0.8 mm, TR = 2,500 ms, TE = 1.81, 3.60, 5.39, and 7.18 ms, TI = 1,000 ms, flip-angle = 8°, matrix 300 × 320 × 208, 208 slices, in-plane generalized auto-calibrating partial parallel acquisition (GRAPPA) acceleration = 2. T2w sampling perfection with application-optimized contrasts using different flip angle evolution sequence (SPACE) parameters: voxel size = 0.8 mm, TR=3,200 ms, TE=564 ms, matrix=300 x 320 x 208, 208 slices, in-plane GRAPPA acceleration = 2. Backup, rapid T1w structural images were also obtained using a multi-echo MPRAGE sequence^74^: voxel size = 1.2 mm, TR = 2,200 ms, TE = 1.57, 3.39, 5.21, 7.03 ms, TI = 1,100 ms, flip-angle = 7°, matrix 192 x 192 x 144, in-plane GRAPPA acceleration = 4.

#### Task-based fMRI Data

Extensive task-based fMRI data were collected to explore functional response properties.^19^ The identical sequence was used for both the task runs and the resting-state fixation runs, ensuring the spatial alignment of the estimated networks from both types of acquisition within each individual. In this study, the task runs were reanalyzed to estimate precision brain networks using standard functional connectivity MRI (fcMRI) network analyses.

#### Task Paradigms

##### Tasks Used for Estimating Matrices

In the original study design, fMRI data were acquired during a resting-state fixation task (FIX) to be used for network estimation; independent data were acquired during active task conditions to probe functional response properties.^19^ Here the task-based data were reanalyzed to explore whether task-based data can robustly and precisely estimate brain networks using standard within-individual fcMRI network analysis. Detailed descriptions of the tasks including exclusions are provided in Du et al.^19^ Tasks reanalyzed here for functional connectivity analysis included a motor (MOT) task designed to estimate somatomotor topography; an Episodic Projection (EPRJ) task that encouraged participants to remember and imagine the future; a visual oddball detection (ODDBALL) task where participants detected salient, uncommon targets among distractors; a working memory (NBACK) task designed to study cognitive control under memory load; and a visual retinotopic stimulation – meridians / eccentricity task (VISME) designed to map retinotopy.

##### Tasks Used for Test-Retest Reliability and Between-Task Similarity Estimates

The available data allowed for three of the acquisition types to be further divided within each participant into independent test and retest samples yielding six distinct datasets (FIX1, FIX2, MOT1, MOT2, EPRJ1, and EPRJ2), all with equivalent amounts of data (Table S2). To ensure comparability, the MOT and EPRJ runs were trimmed to 410 volumes (6 min 50 sec), exactly matching the amount of data in FIX runs. These six matched datasets enabled contrasts between acquisition types (FIX1 versus MOT2) to be compared to test-retest estimates within the same acquisition type (FIX1 versus FIX2, MOT1 versus MOT2). In this manner, the similarity between matrices of data acquired within the same tasks could serve as a reference for contrasts between matrices of different acquisition types. In addition, having two independent estimates of matrices for each acquisition type allowed us to explore whether any acquisition types generally yielded more reliable estimates. For example, it could be the case that resting-state fixation acquisitions generally yield more similar matrices than matrices derived from the task-based acquisitions.

##### Tasks Used for the Construct Validity Test of Functional Specificity

Three critical tasks were further used to measure functional response within independently defined networks and regions (Sentence Processing, SENT; Theory-of-Mind, ToM; Episodic Projection, EPRJ). Given their importance, these three tasks are described in more detail below. Comprehensive task descriptions, contrast details, and code for all tasks are openly available (https://dataverse.harvard.edu/dataset.xhtml?persistentId=doi:10.7910/DVN/AVB4BW).

The Sentence Processing (SENT) task examined domain-specialized processes related to word and phrase-level meaning.^56,75^ Participants passively read real sentences or pronounceable nonword strings. After each word or nonword string, a cue appeared for 0.50 sec, prompting participants to press a button with their right index finger. Word and nonword strings were presented in blocks of three strings. Extended fixation blocks appeared at the start of each run and after every fourth string block. The comparison of interest was the contrast between sentence blocks and non-word blocks to activate the LANG network.

The Theory-of-Mind (ToM) tasks investigated domain-specialized processes associated with understanding other people’s mental states.^76–79^ In the False Belief paradigm, participants read brief stories describing a protagonist with a false belief (False Belief condition) or a physical scene (False Photo condition). In the control False Photo condition, participants indicated whether the story was true or false. In the Pain paradigm, stories described emotionally painful situations (Emo Pain condition) and were contrasted with control stories involving physical pain (Phys Pain condition). At the end of each story, participants rated the level of emotional / physical pain experienced by the protagonist during the question period. Stimuli never repeated. The comparison of interest combined the False Belief versus False Photo and Emo Pain versus Phys Pain contrasts extending from DiNicola et al.^18^

The Episodic Projection (EPRJ) task examined domain-specialized processes related to remembering the past and imagining the future (prospection).^18,80^ In the target task conditions, the participants read scenarios that oriented them to either a past (Past Self) or a future (Future Self) situation, and simultaneously answered questions about the scenarios. The similarly structured control condition asked the participants about a present situation (Present Self). All scenarios were unique. The comparison of interest combined the Past Self versus Present Self and Future Self versus Present Self contrasts extending from DiNicola et al.^18^

##### Tasks Used for Simultaneous Network Estimation and Task Response Extraction

The visual oddball detection (ODDBALL) task investigated detection of transient responses to salient, visual uncommon oddball targets compared to irrelevant and distracting nontargets.^81^ Participants were rapidly presented with uppercase Os and Ks, which appeared in either black or red. Participants pressed a button with their right index finger whenever a red K appeared, and withheld their responses to all the other letter-color combinations. Within each run, target red Ks made up 10% of the trials, lure red Os 10%, and both distractor black Ks and distractor black Os each accounted for 40% of the trials. The comparison of interest was the contrast between target red Ks versus all other trials.

The NBACK task examined working memory under load. Participants were sequentially presented visual letters one at a time and asked to compare the current letter to the previously presented letters indicated when a letter repeated across two intervening trials.^82–84^ Participants pressed a button when the current letter matched the letter presented two trials before. The comparison of interest contrasted the 25-sec blocks of continuous 2-Back trials with intervening 22-sec blocks of visual fixation.

### QUANTIFICATION AND STATISTICAL ANALYSES

#### MRI Data Processing and Surface Registration

The data were processed using the openly available iProc processing pipeline that preserves spatial details by minimizing blurring and interpolations (described in detail in Du et al.^19^ and Braga et al.^45^; relevant methods are repeated here). For resting-state fixation data, the processed data were taken directly from Du et al.^19^ For task-based data, the data were reprocessed in this study using the same processing steps as for resting-state fixation data.

Data were interpolated to a 1-mm isotropic T1w native-space atlas (with all processing steps composed into a single interpolation) that was then projected using FreeSurfer v6.0.0 to the fsaverage6 cortical surface (40,962 vertices per hemisphere; Fischl et al.^85^). Four transformation matrices were calculated: 1) a motion correction matrix for each volume to the run’s middle volume [linear registration, 6 degrees of freedom (DOF); MCFLIRT, FSL], 2) a matrix for field-map-unwarping the run’s middle volume, correcting for field inhomogeneities caused by susceptibility gradients (FUGUE, FSL), 3) a matrix for registering the field-map-unwarped middle BOLD volume to the within-individual mean BOLD template (12 DOF; FLIRT, FSL), and 4) a matrix for registering the mean BOLD template to the participant’s T1w native-space image which was resampled to 1.0 mm isotropic resolution (6 DOF; using boundary-based registration, FreeSurfer). The individual-specific mean BOLD template was created by averaging all field-map-unwarped middle volumes after being registered to an upsampled 1.2 mm and unwarped mid-volume template (an interim target, selected from a low motion run, typically acquired close to a field map).

For resting-state fixation runs and task-based runs used for functional connectivity analysis, confounding variables including six head motion parameters, whole-brain, ventricular and deep cerebral white matter signals, and their temporal derivatives were calculated from the BOLD data in T1w native space. The signals were regressed out from the BOLD data with 3dTproject, AFNI.^67,68^ The residual BOLD data were then bandpass filtered at 0.01–0.1-Hz using 3dBandpass, AFNI.^67,68^ For task-based runs used for standard task contrast analysis, only whole-brain signal was regressed out (see DiNicola et al.^18^). The data were then resampled from T1w native-space atlas to the fsaverage6 standardized cortical surface mesh using trilinear interpolation (featuring 40,962 vertices per hemisphere; Fischl et al.^85^) and then surface-smoothed using a 2-mm full-width-at-half-maximum (FWHM) Gaussian kernel.

#### Estimation of Functional Connectivity Matrices

Functional connectivity was estimated by calculating Pearson correlations between time series from pairs of brain regions, using Fisher’s *z*-transformed values for computations. The functional connectivity maps were averaged across runs to yield a single best estimate of the within-individual functional connectivity map. For resting-state fixation data, this process required no additional steps after processing to calculate the correlations. For task-based data, task structure was removed to allow functional connectivity to be performed on the intrinsic fluctuations unrelated to task events (e.g., trials and blocks). Extending from Fair et al.,^23^ we employed regression to remove the task structure from the fMRI data (see also Cole et al.^25,26^). Specifically, we used residualized data following standard task-based general linear model (GLM) analysis as applied in Du et al.^19^ and input the resulting task-regressed data (residuals) into the standard fcMRI network analysis pipelines. The benefit of this approach is that it is straightforward and allows use of residualized data that is routinely generated from standard task-based processing pipelines without any additional steps.

#### Estimation of Similarity Between Matrices

The broad question of this paper is whether functional connectivity can utilize task data to yield results and network estimates that are equivalent in practice to the traditional use of resting-state fixation data. The first set of analyses examined the equivalence of the correlation matrices directly to determine how similar or different they are when derived from data acquired under different task conditions.

To quantify matrix similarity, we constructed a 1,175 x 1,175 functional connectivity matrix based on 1,175 regions-of-interest (ROIs) uniformly distributed across the two hemispheres.^63,86^ The linearized upper triangles of these symmetrical matrices were used as inputs for calculating similarity values across matrices derived from distinct data sets and following distinct processing choices. For each task, functional connectivity matrices were calculated for each run and mean averaged to produce a stable functional network matrix, which was subsequently used to calculate the similarity matrix between independent data sets from the same acquisition types and between acquisition types (e.g., FIX1 versus FIX2, FIX1 versus MOT2, MOT1 versus MOT2). The similarity value was directly examined by calculating Pearson correlation among the linearized upper triangles of functional connectivity matrices, resulting in a second order “similarity matrix” (see also Gratton et al.^34^). For statistical analyses, similarity values were Fisher’s *z*-transformed, with results converted back to *r* values for reporting.

As a control and to illustrate the importance of conducting analyses within the idiosyncratic anatomy of the individual participant, we also compared the matrices between individuals rather than within individuals. As will be demonstrated, between-individuals correlations were markedly attenuated reinforcing the importance of conducting analyses within the anatomy of the individual.

#### Within-Individual Precision Network Estimates of the Cerebral Cortex

We employed the 15-network MS-HBM previously used by Du et al.^19^ and DiNicola et al.^59^ to estimate networks (derived from the approach developed by Kong et al.^63^). This MS-HBM estimation was applied in small groups, mirroring our previous procedure (see Du et al.^19^ for details). 4 out of 15 of the original participants were excluded due to missing task data, which led to the reanalysis of resting-state fixation data to align participant groupings. Resting-state fixation and task-regressed data were used as inputs in the MS-HBM to estimate precision brain networks for each individual, employing the same standard fcMRI network analysis as outlined in Du et al.^19^ The networks were estimated with separate models for the resting-state acquisitions and for the task acquisitions.

First, the functional connectivity profile of each vertex on the fsaverage6 cortical surface was calculated based on its functional connectivity to 1,175 ROIs uniformly distributed across the fsaverage surface.^86^ For each run of task-based data, Pearson’s correlation coefficients between the fMRI time series at each vertex (40,962 vertices / hemisphere) and the 1,175 ROIs were computed. The resulting 40,962 x 1,175 correlation matrix was then binarized by keeping the top 10% of the correlations to obtain the functional connectivity profiles.^86^ Next, the expectation-maximization algorithm for estimating parameters in the MS-HBM was initialized with a 15-network group-level parcellation, ‘DU15NET-Prior’, derived from a subset of the HCP S900 data release and used to generate within-individual precision network estimates.^19^

After obtaining network estimates separately for resting-state fixation and task-based acquisitions, we examined correspondence using qualitative as well as quantitative procedures. Qualitative correspondence was visualized by comparing the network assignments visually on the fully inflated flatmaps so that all similarities and differences could be appreciated. Quantitative correspondence was computed as the percentage of overlap of the assignment of every individual network for each participant. This is a conservative estimate because there are 15 possible network assignments for every vertex. Positive overlap was recorded only when the vertex was assigned to the exact same network between the two compared maps. Moreover, we did not exclude any vertices. Overlap misses, even in regions of low signal-to-noise, penalize the estimates of correspondence. To anchor the interpretation of the estimate, we also compute quantitative similarity for identically acquired resting-state fixation data.

#### Model-Free Seed-Region Tests of Spatial Correspondence

Winner-take-all assignments to individual networks do not always capture the complexity of spatial correlation patterns, given that vertices assigned to one network may nonetheless have correlations with others. As another means to visualize correspondence between network estimates derived from traditional resting-state fixation versus task acquisitions, a further set of seed-region based analyses was performed.

For each participant, a flatmap was created with the outlines of each network derived solely from the resting-state fixation data. Seed regions were then manually placed in posterior medial, parietal, and prefrontal zones for each network. The correlation patterns from these seed regions from the task-regressed data were then visualized in relation to the network borders. That is, the spatial correlation patterns from the task-regressed data were plotted in relation to the network borders estimated from the traditional resting-state fixation data. This is an extreme test because the task-regressed data are independent and unconstrained by any model assumptions.

#### Construct Validity Test of Functional Specificity Using Independent Task Data

To investigate the practical effects of using network estimates derived from task-regressed data, we explored whether the estimated networks could prospectively predict functional response properties in independent tasks collected from separate MRI acquisitions. The logic of this analysis is that network estimates are often used as functional localizers to then predict task responses. For example, the estimate of the LANG network predicts regions preferentially responsive to meaning-based sentence processing tasks (e.g., Braga et al.^87^ and Fedorenko et al.^88^). That is, the networks are hypothesized to localize meaningful biological regions that predict response patterns in additional tasks and experimental contexts.

For this analysis, we generated network estimates for each participant from task-regressed data that included the following tasks: MOTOR, ODDBALL, NBACK and VISME (referred to as “Task Acquisition” network estimates; see Table S2). These task-based network estimates were directly compared to network estimates derived solely from traditional resting-state fixation data (referred to as “Fixation Acquisition” network estimates). These two sets of estimates – the Task Acquisition network estimates and the Fixation Acquisition network estimates – were then used as the basis for testing a triple functional dissociation in independent task data.

There are three well characterized association networks that are juxtaposed with one another across the distributed zones of association cortex that are functionally specialized (DN-A, DN-B, and LANG). These networks are spatially intertwined, making them challenging to separate. Nonetheless, using within-individual precision approaches, they have been reliably identified and functionally dissociated.^18,19,59,87^ This established and robust functional specificity was leveraged here to test the validity of the network estimates that were derived from the task data. Specifically, we asked whether the Fixation Acquisition network estimates could predict functional responses that are preferential for the EPRJ task contrast (where the response in DN-A is greatest), the ToM task contrasts (where the response in DN-B is greatest), and the SENT task contrast (where the response in LANG is greatest). That is, could network estimates derived exclusively from task data predict a known functional triple dissociation in independent data?

For this final test, functional task data (EPRJ, ToM and SENT) were analyzed using the GLM (FEAT, FSL; Smith et al.^65^ and Woolrich et al.^89^) as previously described in Du et al.^19^ GLM outputs included *β*-values for each contrast at each vertex, which were converted to *z*-values within FEAT. For each participant, *z*-value maps from all runs were averaged using *fslmaths* to create a single cross-session map for each task contrast of interest. To quantify the functional response within the independently defined cerebral networks, we calculated the average *z*-value of a specific task contrast for all vertices within each defined network, excluding those vertices directly bordering adjacent networks where assignment confidence was low.^86^ We then calculated the mean *z*-values across runs to obtain a single response value for each network from each participant. The mean *z*-values across participants were then visualized in the bar graphs, along with the standard error of the mean. We repeated the analysis for spatially restricted portions of the cerebral cortex, including lateral prefrontal cortex, temporal association cortex, and medial prefrontal cortex. These regions, in particular temporal cortex and the frontal midline, are zones of particularly challenging between-person spatial variation. In all cases, the networks were defined within individuals based on functional connectivity derived from either the resting-state fixation data (“Fixation Acquisition”) or the separate task-based data (“Task Acquisition”). Critically, the target task data used to test for functional dissociation was independent from the data used to estimate the networks.

#### Within-Individual Precision Estimates of Thalamic Association Zones

As the results will reveal, task-regressed data can be used to estimate individualized networks. An implication of this observation is that previously acquired task data can be reanalyzed and pooled in novel ways. Here we illustrate an extreme variation of such an analysis to tackle the challenging task of mapping association zones of the human thalamus.

Studies of direct anatomical connectivity in the monkey reveal that cortical association zones, including the prefrontal cortex, project to midline thalamic nuclei extending from the medial pulvinar nucleus through the mediodorsal and anterior nuclei.^90–93^ What makes the thalamus challenging to map in the human is its small size and presumed anatomical variability from one person to the next. Given that the cerebellum and striatum maintain partial segregation of association networks (e.g., Saadon-Grosman et al.,^69^ Kosakowski et al.,^70^ Marek et al.,^94^ Xue et al.,^95^ Greene et al.,^96^ and Gordon et al.^97^), the thalamus is expected to also possess parallel (partial) segregation of brain-wide circuits.^53^ Despite this expectation, we have previously failed to reveal segregated functional organization of the thalamus in our large group-based studies (e.g., Yeo et al.^86^) presumably because of spatial blurring. Recent individualized approaches have successfully found the midline thalamus is linked to association networks including the DN and noted spatial variation between people (e.g., see Figure 3 of Greene et al.^96^) suggesting that it might be possible to identify adjacent subregions along the thalamic midline if signal-to-noise ratio (SNR) is increased to a sufficient degree within the idiosyncratic anatomy of the individual.

To expand on this possibility and illustrate a novel opportunity enabled by this analysis strategy, we mapped the association zones of the thalamus by combining all available data (task and resting-state fixation data) from Du et al.^19^ That is, unlike the earlier analyses that compared task-regressed data to traditional resting-state fixation data, here we pooled the two data types to maximize the SNR within each participant. In doing so, the amount of available data was increased to between 47 and 78 runs for P1 to P15 (296 min to 475 min). These data were then randomly shuffled within each participant and assigned to independent Discovery and Replication datasets. To estimate the organization of the association zones of the thalamus, a winner-takes-all assignment of each thalamic voxel was computed for each independent dataset within each of the individual (paralleling the approach of Saadon-Grosman et al.,^69^ Kosakowski et al.,^70^ and Xue et al.^95^ for the striatum and cerebellum). Specifically, functional connectivity was computed between each thalamic voxel to all vertices on the cortical surface. For each thalamic voxel, we identified cerebral cortical vertices exhibiting significant functional connectivity and assigned this voxel to the network with the highest proportion of significant vertices on the cerebral cortex. Note that each voxel in the thalamus had the opportunity to be assigned to any of the 15 cortical networks. To quantify assignment certainty, we also computed a confidence measure for each voxel, defined as the proportion of vertices assigned to the most frequent (winner-takes-all) network relative to the combined proportion for the top two frequent networks. The association zones of the thalamus were then visualized for each individual and also a composite was made to show where, on average, the higher-order association networks were represented in the thalamus.

To provide another means to assess the segregation of the thalamic subregions without a winner-takes-all assumption, we examined whether raw correlations from the thalamus could recapitulate the spatially distinct cortical networks using a model-free seed-region based approach.^69,70,95,98^ Specifically, we computed the functional connectivity patterns generated from thalamic seed regions and, without applying any prior constraints, tested if they could recapitulate the distributed functional connectivity pattern within each network’s boundaries in the cerebral cortex. The unconstrained resulting functional connectivity patterns across the cerebral cortex were visualized using Connectome Workbench.^64^ If a thalamic seed region located within a specific region boundary reproduced the corresponding cortical network pattern, this would support the validity of the network estimates. By contrast, if the resulting cortical functional connectivity maps were nonspecific, fractionated networks or spanned multiple networks, this would challenge the validity of the results. Thus, this model-free seed-based analysis served as a critical validation (control) analysis.

#### Between-Individual Differences in Task Response Extracted from Precision Network Estimates

Reliable measures of between-individual differences in brain activity and their associations with behavioral and genetic traits are critical for basic and clinical neuroscience.^99,100^ We investigated whether the same task data can be concurrently used both to estimate precision networks and to measure task response properties from regions within those networks, potentially reducing the need for separate resting-state fixation scan acquisitions. To address this, we utilized 5 ODDBALL task-regressed runs and 5 resting-state fixation runs for 8 participants with sufficient available data (data again from Du et al.^19^ participants). Each fixation run was trimmed to match the duration of ODDBALL task runs, ensuring an equivalent amount of data between the two datasets.

The resting-state fixation and ODDBALL task-regressed data were independently input into the 15-network MS-HBM model to estimate within-individual precision networks. Following this, we quantitatively evaluated the ODDBALL response level from the same ODDBALL task runs within specific networks – including CG-OP, SAL / PMN, DN-A and DN-B networks in both sets of within-individual precision networks. The oddball effect is expected to increase response in CG-OP and SAL / PMN networks, and robustly decrease the response in the DN-A and DN-B networks (see Figures 25 and 26 in Du et al.^19^). Our central hypothesis was that task-regressed data, when matched in quantity to resting-state fixation data, would yield between-individual differences in task responses that are equivalent to those derived from resting-state fixation data, demonstrating that within-individual idiosyncratic anatomical differences are robustly captured using either acquisition. If successful, this task design offers a novel strategy to exclusively use task-based data simultaneously to obtain both within-individual precision network estimates and network-level functional response quantification.

#### Precision Network Estimation and Task Extraction from a Single Low Burden MRI Session

To demonstrate that task-based data can be used to simultaneously obtain precision network estimates and extract task responses, we replicated a well-established task effect from the literature in a single ∼1 h session of MRI data. Working memory tasks, particularly the NBACK task under memory load, have repeatedly yielded a robust response in a distributed association network generally referred to as the frontoparietal control network or multiple-demand network.^59,101,102^ In Du et al.^19^ this network was isolated, referred to as FPN-A, using repeated acquisitions of resting-state fixation runs. The response of the network was then extracted from independent task runs where participants performed the NBACK task. A strong preferential response was found in FPN-A.

Here we acquired 10 runs of the NBACK task in one session for a single participant with 5 runs using letter stimuli similar to Du et al.^19^ and 5 runs using picture stimuli. Task-regressed data from all runs were input into the 15-network MS-HBM model to automatically estimate within-individual precision networks. The mean response from each run for each network was then extracted for the NBACK task and plotted in direct comparison to the original data acquired by Du et al.^19^ The analyses thus utilized only a single (modest burden) session of task data with fully automated processing to test, as a proof-of-concept, whether a preferential response in a specific higher-order association network can be efficiently extracted. Data were processed using the same procedures as above with the addition of motion correction (see Sun et al.^103^ for description).

## Supplementary Material

Supplementary Material

Supplemental information can be found online at https://doi.org/10.1016/j.neuron.2025.08.029.

## Figures and Tables

**Table:** KEY RESOURCES TABLE

REAGENT or RESOURCE	SOURCE	IDENTIFIER
Software and algorithms		

Connectome Workbench	Marcus et al.^64^	www.humanconnectome.org
MATLAB	MathWorks	www.mathworks.com
FSL	Smith et al.^65^	https://fsl.fmrib.ox.ac.uk/fsl/fslwiki/
FreeSurfer	Fischl^66^	http://surfer.nmr.mgh.harvard.edu
AFNI	Cox^67,68^	http://afni.nimh.nih.gov/

## Highlights

- Whole-brain within-individual networks can be estimated from arbitrary task data
- Network estimates from task data can be used to localize functionally distinct regions
- Task and resting-state data can be pooled to increase statistical power
- The same task data can be used to estimate networks and extract functional responses
