# Supplementary material for: Within-individual precision mapping of brain networks exclusively using task data
Source: Neuron. Author manuscript; Available in PMC 2026 Feb 25. (PMC12935283; doi:10.1016/j.neuron.2025.08.029)
Supplement: Supplementary Material [file NIHMS2136304-supplement-Supplementary_Material.pdf]

Neuron, Volume *113*

Supplemental information

Within-individual precision mapping

of brain networks exclusively using task data

Jingnan Du, Vaibhav Tripathi, Maxwell L. Elliott, Joanna Ladopoulou, Wendy Sun, Mark C. Eldaief, and Randy L. Buckner

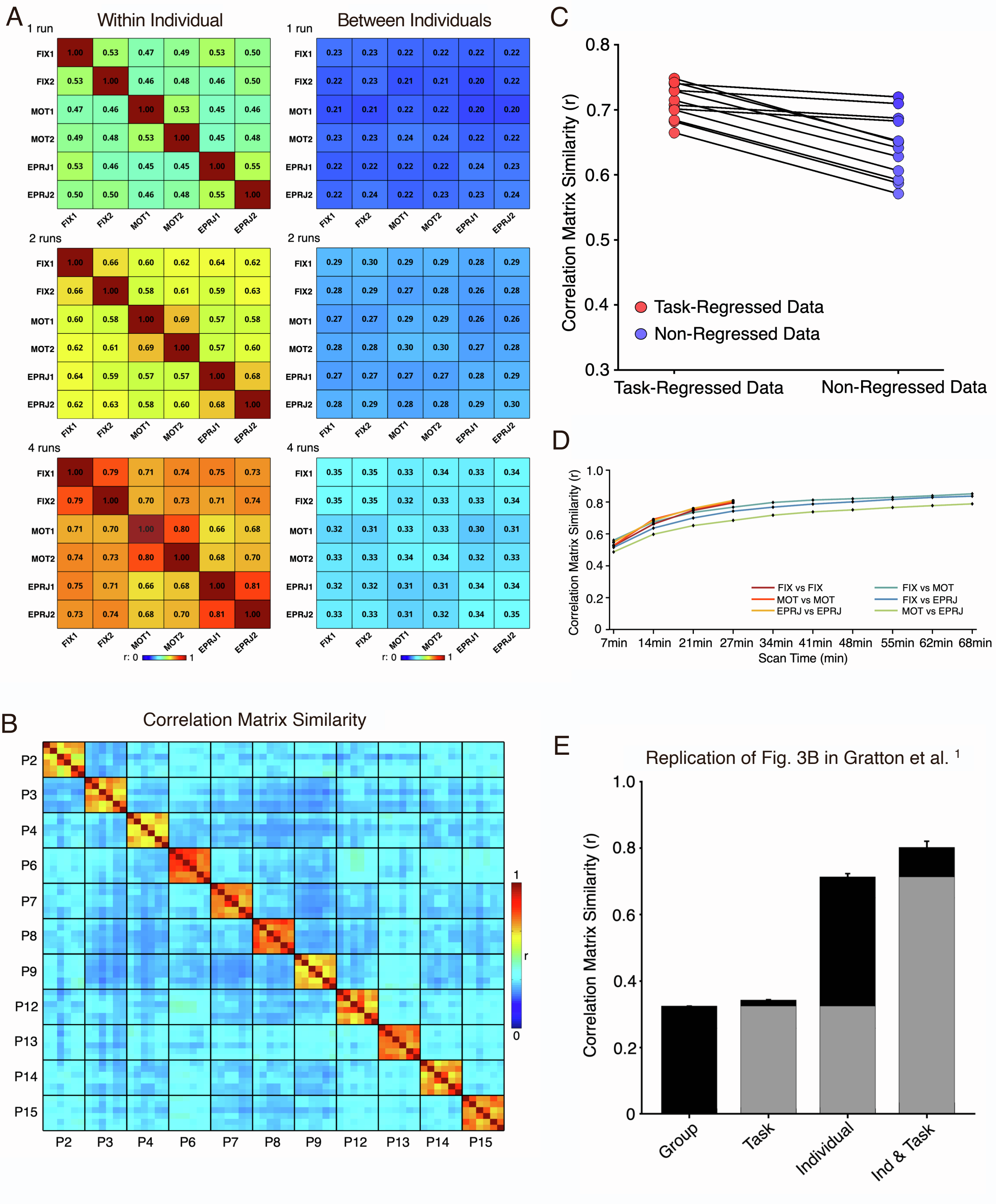

A
Within Individual
Between Individuals
1 run
FIX1 1.00 0.53 0.47 0.49 0.53 0.50
FIX2 0.53 1.00 0.46 0.48 0.46 0.50
MOT1 0.47 0.46 1.00 0.53 0.45 0.46
MOT2 0.49 0.48 0.53 1.00 0.45 0.48
EPRJ1 0.53 0.46 0.45 0.45 1.00 0.55
EPRJ2 0.50 0.50 0.46 0.48 0.55 1.00
FIX1 FIX2 MOT1 MOT2 EPRJ1 EPRJ2
1 run
FIX1 0.23 0.23 0.22 0.22 0.22 0.22
FIX2 0.22 0.23 0.21 0.21 0.20 0.22
MOT1 0.21 0.21 0.22 0.22 0.20 0.20
MOT2 0.23 0.23 0.24 0.24 0.22 0.22
EPRJ1 0.22 0.22 0.22 0.22 0.24 0.23
EPRJ2 0.22 0.24 0.22 0.23 0.23 0.24
FIX1 FIX2 MOT1 MOT2 EPRJ1 EPRJ2
2 runs
FIX1 1.00 0.66 0.60 0.62 0.64 0.62
FIX2 0.66 1.00 0.58 0.61 0.59 0.63
MOT1 0.60 0.58 1.00 0.69 0.57 0.58
MOT2 0.62 0.61 0.69 1.00 0.57 0.60
EPRJ1 0.64 0.59 0.57 0.57 1.00 0.68
EPRJ2 0.62 0.63 0.58 0.60 0.68 1.00
FIX1 FIX2 MOT1 MOT2 EPRJ1 EPRJ2
2 runs
FIX1 0.29 0.30 0.29 0.29 0.28 0.29
FIX2 0.28 0.29 0.27 0.28 0.26 0.28
MOT1 0.27 0.27 0.29 0.29 0.26 0.26
MOT2 0.28 0.28 0.30 0.30 0.27 0.28
EPRJ1 0.27 0.27 0.27 0.27 0.28 0.29
EPRJ2 0.28 0.29 0.28 0.28 0.29 0.30
FIX1 FIX2 MOT1 MOT2 EPRJ1 EPRJ2
4 runs
FIX1 1.00 0.79 0.71 0.74 0.75 0.73
FIX2 0.79 1.00 0.70 0.73 0.71 0.74
MOT1 0.71 0.70 1.00 0.80 0.66 0.68
MOT2 0.74 0.73 0.80 1.00 0.68 0.70
EPRJ1 0.75 0.71 0.66 0.68 1.00 0.81
EPRJ2 0.73 0.74 0.68 0.70 0.81 1.00
FIX1 FIX2 MOT1 MOT2 EPRJ1 EPRJ2
r: 0 1
4 runs
FIX1 0.35 0.35 0.33 0.34 0.33 0.34
FIX2 0.35 0.35 0.32 0.33 0.33 0.34
MOT1 0.32 0.31 0.33 0.33 0.30 0.31
MOT2 0.33 0.33 0.34 0.34 0.32 0.33
EPRJ1 0.32 0.32 0.31 0.31 0.34 0.34
EPRJ2 0.33 0.33 0.31 0.32 0.34 0.35
FIX1 FIX2 MOT1 MOT2 EPRJ1 EPRJ2
r: 0 1
C
Correlation Matrix Similarity (r)
0.8 0.7 0.6 0.5 0.4 0.3
Task-Regressed Data
Non-Regressed Data
Task-Regressed Data
Non-Regressed Data
D
Correlation Matrix Similarity (r)
1.0 0.8 0.6 0.4 0.2 0
FIX vs FIX
MOT vs MOT
EPRJ vs EPRJ
FIX vs MOT
FIX vs EPRJ
MOT vs EPRJ
7min 14min 21min 27min 34min 41min 48min 55min 62min 68min
Scan Time (min)
B
Correlation Matrix Similarity
P2 P3 P4 P6 P7 P8 P9 P12 P13 P14 P15
1
r
0
P2 P3 P4 P6 P7 P8 P9 P12 P13 P14 P15
E
Replication of Fig. 3B in Gratton et al. 1
Correlation Matrix Similarity (r)
1.0 0.8 0.6 0.4 0.2 0
Group
Task
Individual
Ind & Task

Figure S1

Figure S1. (Related to Figure 2) Functional correlation matrices estimated from task-regressed data are highly similar to those estimated from resting-state fixation data, with the amount of data being the largest factor affecting similarity.

Panels (**A**) The similarity estimates between pairs of functional correlation matrices are presented for matched-length data acquired during resting-state fixation (FIX), motor (MOT) and episodic projection (EPRJ) task runs. For each acquisition type, two independent datasets allowed test-retest similarity within an acquisition type (e.g., FIX1 versus FIX2) to be contrasted with similarity between acquisition types (e.g., FIX1 versus EPRJ1). Within-individual similarity was calculated within each participant and then mean averaged across all participants (Left). Between-individuals similarity was calculated between each pair of participants and then averaged across all pairs (Right). The rows show similarity values for different amounts of data (1, 2, and 4 runs). The colors reflect their correlation strength as noted by the legend below. What is notable is that the correlation values between acquisition types were almost as high as the values within acquisition types for all amounts of data, suggesting that the underlying region-to-region correlation structure is largely, but not entirely, preserved across acquisition conditions. The within-individual similarity values are much higher than the between-individuals similarity values. The primary factor impacting the similarity between functional correlation matrices within individuals was the amount of data. (**B**) High similarity between task-regressed and resting-state fixation correlation matrices is observed in each individual. The similarity estimates between pairs of functional correlation matrices are presented for matched-length data acquired during resting-state fixation (FIX), motor (MOT) and episodic projection (EPRJ) task runs within and between individuals. For each acquisition type, two independent datasets allowed test-retest similarity within an acquisition type (e.g., FIX1 versus FIX2) to be contrasted with similarity between acquisition types (e.g., FIX1 versus EPRJ1). The colors reflect their correlation strength as noted by the legend on the right. What is notable is that the correlation values between acquisition types were almost as high as the values within acquisition types for all amounts of data, suggesting that the underlying region-to-region correlation structure is largely, but note entirely, preserved across acquisition conditions. The within-individual similarity values are much higher than the between-individuals similarity values. (**C**) Regressing out task structure increases the similarity of correlation matrices between datasets from different task types (FIX1, FIX2, MOT1, MOT2, EPRJ1, EPRJ2; 4 runs for each) within individuals. Each dot represents the average similarity for a pair of datasets across individuals, with lines connecting the same pair before and after regression. Task-regressed data show consistently higher similarity values for all pairs. (**D**) Similarity between correlation matrices

increases with more data. The similarity between correlation matrices is computed both between and within tasks, including resting-state fixation (FIX), motor (MOT), and episodic projection (EPRJ) tasks, within the same individuals. Within-task similarity is plotted for independent test and retest datasets from the same individuals. The similarity between functional correlation matrices derived from independent data improves with increasing amounts of data. Additionally, the similarity of the correlation matrices estimated from task-regressed data to the resting-state fixation data is almost as high as any of the data types to themselves. This indicates that functional correlation matrices from task data have similar reliability to those derived from traditional resting-state fixation data. The variance in correlation matrices remains largely consistent regardless of the task performed during data acquisition. (**E**) Individual-level factors account for most variance in the similarity of correlation matrices, with modest contributions from task state. Following Gratton et al. [1], bar heights represent the similarity of correlation matrices under four comparison conditions: Group (between different individuals and different acquisition types), Task (between different individuals but within the same acquisition type), Individual (within the same individual but across different acquisition types), Ind & Task (within the same individual and the same acquisition type). Similarity is the lowest between different individuals, increases slightly when comparing different individuals within the same acquisition type, is substantially higher for the same individual across acquisition types, and reaches its peak within the same individual and acquisition type. These results indicate that individual differences account for the majority of the variance, with modest state-level (task) effects. Error bars represent standard error across runs. These findings replicate Gratton et al. [1].

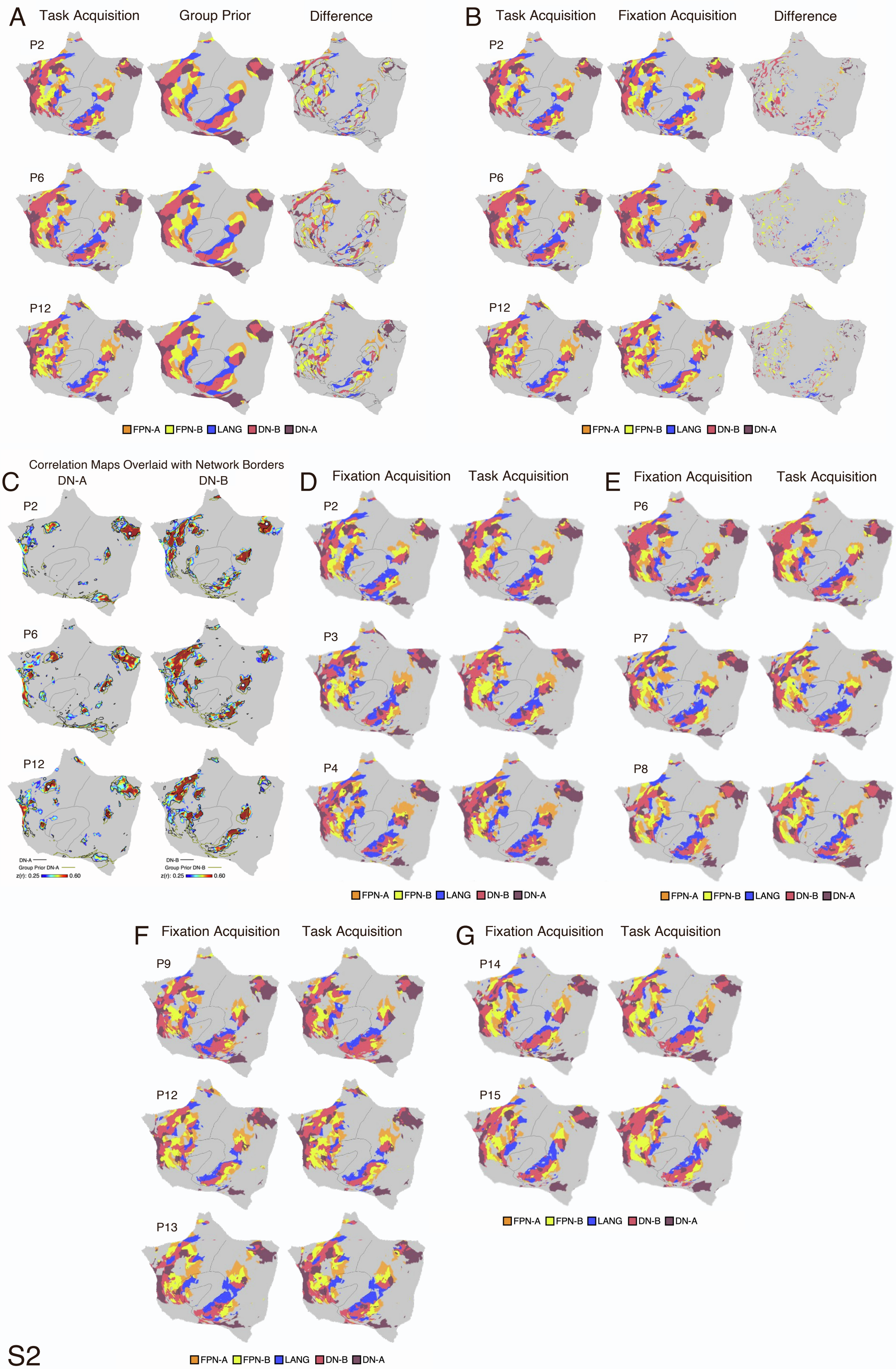
A
Task Acquisition
Group Prior
Difference
P2
P6
P12
FPN-A FPN-B LANG DN-B DN-A
B
Task Acquisition
Fixation Acquisition
Difference
P2
P6
P12
FPN-A FPN-B LANG DN-B DN-A
C
Correlation Maps Overlaid with Network Borders
DN-A
DN-B
P2
P6
P12
DN-A
Group Prior DN-A
z(r): 0.25 0.60
DN-B
Group Prior DN-B
z(r): 0.25 0.60
D
Fixation Acquisition
Task Acquisition
P2
P3
P4
FPN-A FPN-B LANG DN-B DN-A
E
Fixation Acquisition
Task Acquisition
P6
P7
P8
FPN-A FPN-B LANG DN-B DN-A
F
Fixation Acquisition
Task Acquisition
P9
P12
P13
FPN-A FPN-B LANG DN-B DN-A
G
Fixation Acquisition
Task Acquisition
P14
P15
FPN-A FPN-B LANG DN-B DN-A

Figure S2

Figure S2. (Related to Figure 2) Individual-specific brain network estimates are highly consistent between task-regressed and resting-state fixation data, capturing idiosyncratic spatial features not accounted for by group-based parcellations.

Panels (**A**) Side-by-side comparison of within-individual network estimates and group prior parcellation in three representative participants. Each row displays data from a separate participant (P2, P6, and P12). The first column shows individual-specific network estimates derived from task-regressed data. The second column displays the group prior parcellation used in the MS-HBM model. The third column presents 'Difference' maps, calculated by comparing the individual-specific estimates to the group prior, highlighting the idiosyncratic features of each individual's network organization that are not captured by the group-derived atlas. These results serve to visualize the substantial individual differences in network organization that are not accounted for by the group prior. (**B**) Within-individual network estimates across two independent acquisitions highlight their minimal differences. Each row depicts results from a representative participant (P2, P6, and P12). The first column shows individualized network maps estimated from task-regressed data (Task Acquisition), and the second column shows network maps from resting-state fixation data (Fixation Acquisition). The third column presents 'Difference' maps, highlighting regions where network assignments differ between the two acquisitions for the same individual. The minimal differences observed in the third column, compared to group-based comparisons in Figure S2A, demonstrate strong consistency of individual-specific network features across independent datasets. These findings suggest that the individualized parcellation methods preserve unique spatial network features within individuals. (**C**) Comparison of individual-specific correlation maps with group-defined network boundaries. For three representative participants (P2, P6, P12), individual-specific task-regressed correlation maps are displayed with network boundaries overlaid: black outlines indicate individual-specific MS-HBM network estimates defined by independent resting-state fixation data within the same individual, while green outlines indicate network boundaries of the group prior from the MS-HBM model. Correlation maps are shown for two representative networks (DN-A and DN-B). The correlation maps align well with the individual-specific network boundaries across multiple cortical zones, whereas group prior network boundaries fail to capture the idiosyncratic correlation patterns. These findings highlight that idiosyncratic spatial correlation properties within individuals are not fully captured by group-level parcellations. (**D-G**) Network estimates are similar between resting-state fixation and task data acquisitions. Higher-order networks from the 15-network MS-HBM estimates are displayed for all 11 participants for data acquired using traditional resting-state fixation data (Left, Fixation Acquisition) versus exclusively using task-regressed data (Right, Task

Acquisition). The data yielding the network estimates in the left columns are independent of the data used in the right columns. The parallel interdigitated distributed networks, FPN-A, FPN-B, LANG, DN-B, and DN-A, are displayed, allowing for direct comparison of both the broad distributed patterns and idiosyncratic local details. The estimated networks are strikingly similar across both datasets, capturing idiosyncratic, small regions consistently. These findings suggest that task-regressed data can effectively estimate brain networks. FPN-A, Frontoparietal Network-A; FPN-B, Frontoparietal Network-B; DN-A, Default Network-A; DN-B, Default Network-B; LANG, Language.

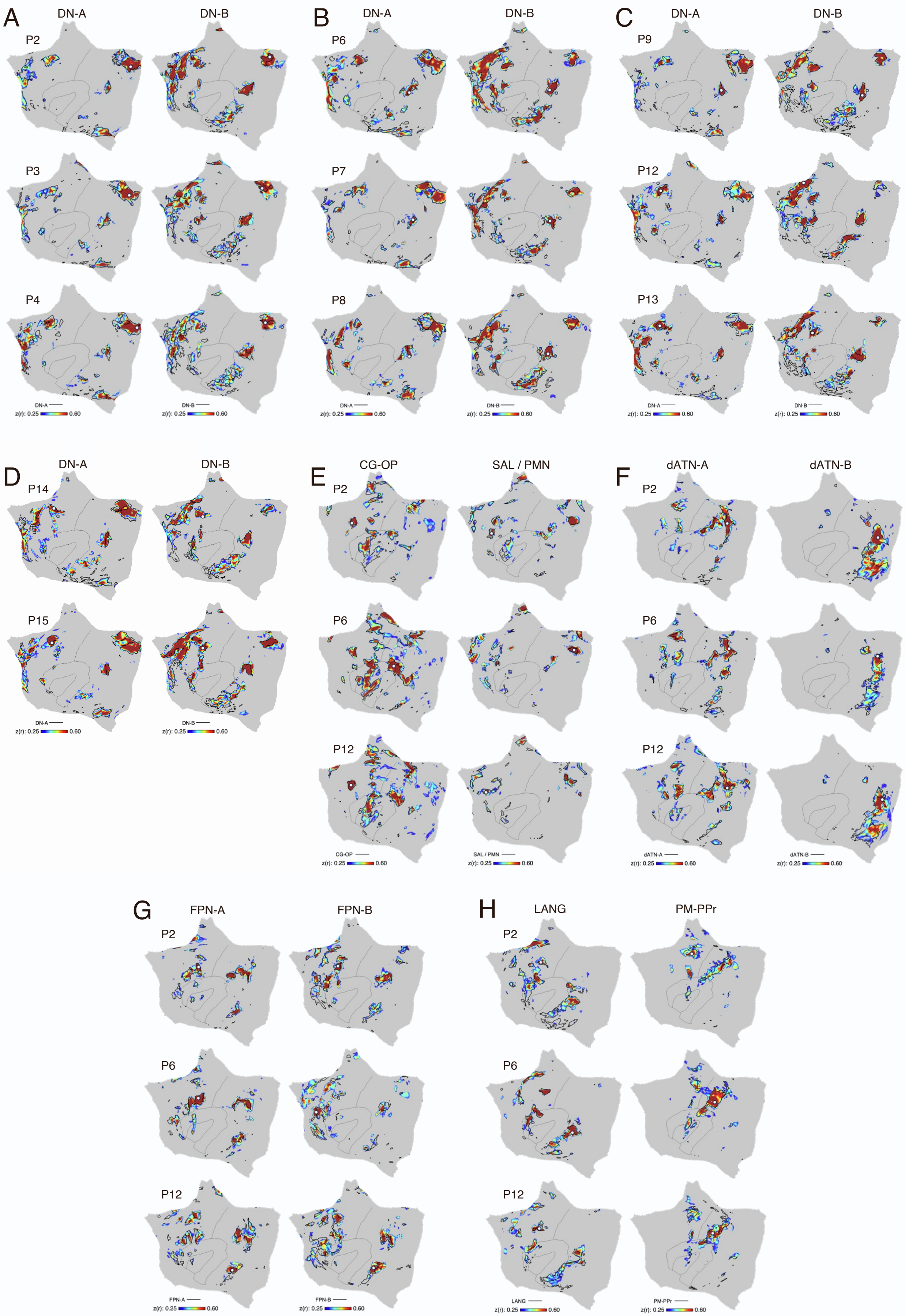
A
DN-A
DN-B
P2
P3
P4
B
DN-A
DN-B
P6
P7
P8
C
DN-A
DN-B
P9
P12
P13
D
DN-A
DN-B
P14
P15
E
CG-OP
SAL / PMN
P2
P6
P12
F
dATN-A
dATN-B
P2
P6
P12
G
FPN-A
FPN-B
P2
P6
P12
H
LANG
PM-PPr
P2
P6
P12
z(r): 0.25
0.60

Figure S3

Figure S3. (Related to Figure 3) Model-free seed-region correlation maps confirm spatial correspondence between resting-state fixation and task data acquisitions for higher-order association networks.

Panels (**A-D**) Spatial correspondence between resting-state fixation and task data acquisitions for DN-A and DN-B. The correlation maps from individual seed regions placed within networks DN-A and DN-B are displayed for all 11 participants. These correlation maps, based exclusively on task-regressed functional connectivity, are plotted as z(r) with the color scale at the bottom. Black outlines show the boundaries of individual-specific networks estimated from the independent resting-state fixation data within the same individual. White-filled circles mark the seed region locations. The positions of the seed regions are moved across association zones between participants to demonstrate that the full distributed extent of DN-A and DN-B can be effectively generated from many component regions. The network boundaries from resting-state fixation data align well with the spatial correlation properties of the task-regressed data establishing correspondence between resting-state fixation and task-regressed data. (E-H) Spatial correspondence between resting-state fixation and task data acquisitions for other higher-order association networks. The correlation maps from individual seed regions placed within networks CG-OP, SAL / PMN, dATN-A, dATN-B, FPN-A, FPN-B, LANG and PM-PPr are displayed for the three representative participants in Figure 3 (P2, P6, P12). These correlation maps, based exclusively on task-regressed functional connectivity, are plotted as z(r) with the color scale at the bottom. Black outlines show the boundaries of individual-specific networks estimated from the independent resting-state fixation data within the same individual. White-filled circles mark the seed region locations. The network boundaries from resting-state fixation data align well with the spatial correlation properties of the task-regressed data establishing correspondence between resting-state fixation and task-regressed data. DN-A, Default Network-A; DN-B, Default Network-B; CG-OP, Cingulo-Opercular; SAL / PMN, Salience / Parietal Memory Network; dATN-A, Dorsal Attention-A; dATN-B, Dorsal Attention-B; FPN-A, Frontoparietal Network-A; FPN-B, Frontoparietal Network-B; LANG, Language; PM-PPr, Premotor-Posterior Parietal Rostral.

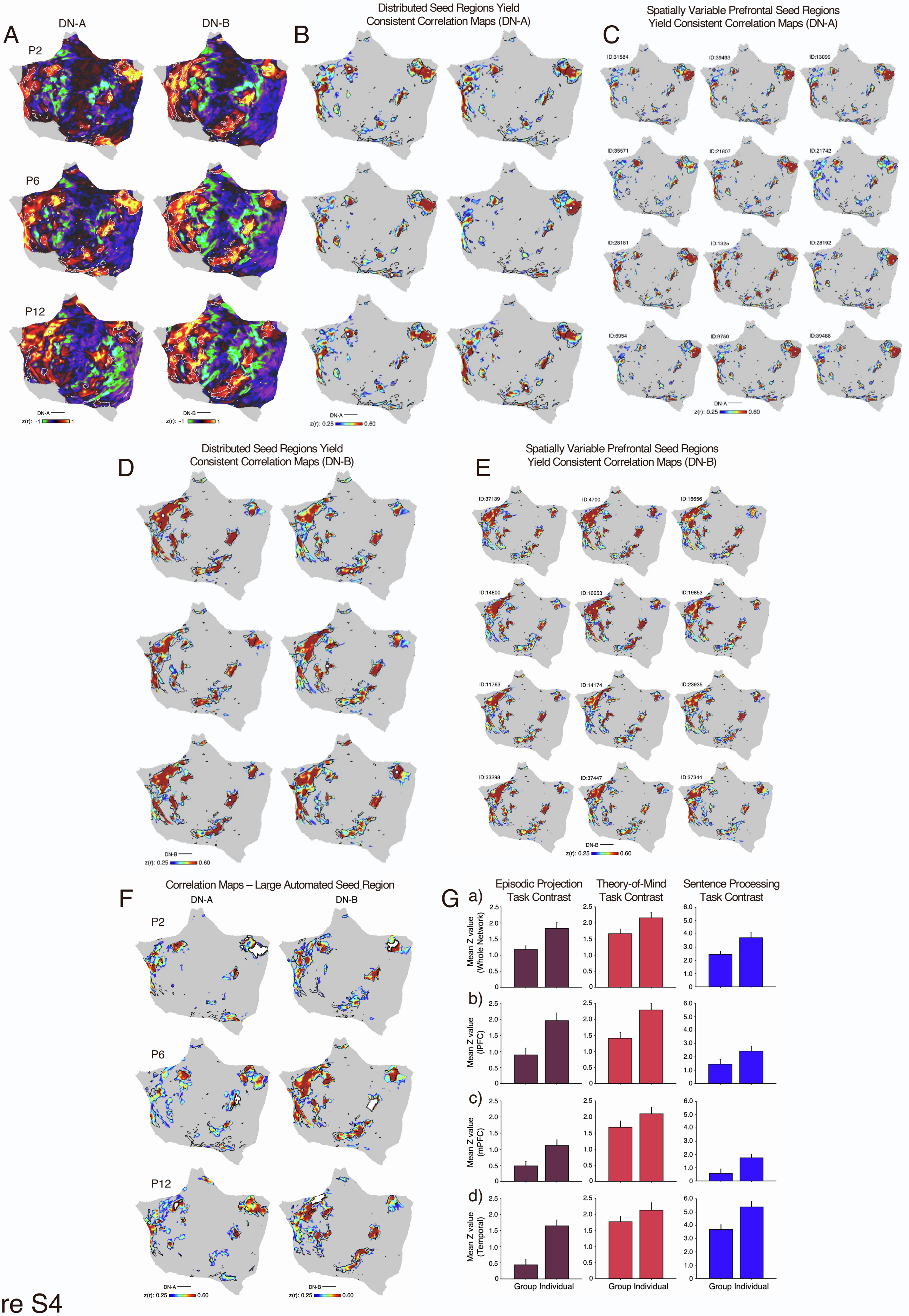
A
DN-A
DN-B
P2
P6
P12
z(r): -1 1
B
Distributed Seed Regions Yield
Consistent Correlation Maps (DN-A)
z(r): 0.25 0.60
C
Spatially Variable Prefrontal Seed Regions
Yield Consistent Correlation Maps (DN-A)
ID:31584
ID:39493
ID:13099
ID:35571
ID:21807
ID:21742
ID:28181
ID:1325
ID:28192
ID:6954
ID:9750
ID:39488
D
Distributed Seed Regions Yield
Consistent Correlation Maps (DN-B)
E
Spatially Variable Prefrontal Seed Regions
Yield Consistent Correlation Maps (DN-B)
ID:37139
ID:4700
ID:16656
ID:14800
ID:16653
ID:19853
ID:11763
ID:14174
ID:23935
ID:33298
ID:37447
ID:37344
F
Correlation Maps – Large Automated Seed Region
G
Episodic Projection
Task Contrast
Theory-of-Mind
Task Contrast
Sentence Processing
Task Contrast
a)
b)
c)
d)
Mean Z value
(Whole Network)
(lPFC)
(mPFC)
(Temporal)
Group
Individual

Figure S4

Figure S4. (Related to Figures 3 and 4) Model-free seed-region correlation maps confirm robust and spatially consistent correlation maps across data acquisition and seed placement strategies.

Panels (**A**) Unthresholded model-free seed-region correlation maps confirm spatial correspondence between resting-state fixation and task data acquisitions. Unthresholded correlation maps from individual seed regions placed within networks DN-A and DN-B are shown for three representative participants (P2, P6, and P12), paralleling the analysis in Figure 3. Correlation patterns are based exclusively on task-regressed functional connectivity and are plotted as z(r), using the full color scale range to show both positive and negative correlations. White outlines indicate the boundaries of individual-specific networks that were defined independently from resting-state fixation data using the MS-HBM model. White-filled circles mark the seed region locations. These unthresholded maps provide a complete view of the spatial selectivity of functional connectivity patterns, demonstrating that the underlying correlation patterns were robustly captured by the MS-HBM model. (**B**) Correlation patterns of DN-A remain highly consistent across distributed seed regions. Correlation maps are shown for multiple distributed seed regions placed across multiple regions within the DN-A network boundaries of a representative participant (P6). For each seed region, functional connectivity patterns were estimated using the same task-regressed data and are shown as z(r), with the color scale at the bottom left. Black outlines show the DN-A network boundaries as estimated from independent resting-state fixation data using the MS-HBM model. Despite varying the location of seed regions within DN-A, the resulting correlation patterns are highly consistent and recapitulate the full distributed organization of the network. These results demonstrate the robustness of DN-A functional connectivity estimates to seed region placement. (**C**) Correlation patterns of DN-A remain highly consistent when seed regions are variably placed within the posterior midline. Correlation maps are shown for twelve seed regions placed within the posterior midline portion of the DN-A network for a representative participant P6. Each panel is labeled by vertex ID in fsaverage6 surface space. For each seed region, functional connectivity was estimated using the task-regressed data and is displayed as z(r), with the color scale indicated at the bottom. Black outlines indicate DN-A network boundaries derived from independent resting-state fixation data using the MS-HBM model. Despite variation in the location of each seed region within posterior midline region, the resulting correlation patterns remain highly consistent - demonstrating the robustness of the underlying correlation pattern of the DN-A network to variability in seed region location. (**D**) Correlation patterns for DN-B remain highly consistent across distributed seed regions. Correlation maps are shown for

multiple distributed seed regions placed within the DN-B network boundaries of a representative participant. For each seed region, functional connectivity patterns were estimated using the same task-regressed data and are shown as z(r), with the color scale at the bottom left. Black outlines show the DN-B network boundaries as estimated from independent resting-state fixation data using the MS-HBM model. Despite varying the location of seed regions within DN-B, the resulting correlation patterns are highly consistent and recapitulate the distributed organization of the network. These results demonstrate the robustness of DN-B functional connectivity estimates to seed region placement. (**E**) Correlation patterns for DN-B remain highly consistent even when seed regions are variably placed within prefrontal cortex. Correlation maps are shown for twelve seed regions placed at variable locations within the prefrontal cortex (PFC) of the DN-B network for a representative participant P6. Each panel is labeled by vertex ID in fsaverage6 surface space. For each seed region, functional connectivity was estimated using the task-regressed data and is displayed as z(r), with the color scale indicated at the bottom. Black outlines indicate DN-B network boundaries derived from independent resting-state fixation data using the MS-HBM model. Despite variation in the location of each seed region within prefrontal cortex, the resulting correlation patterns remain highly consistent - demonstrating the robustness of the underlying correlation pattern of DN-B network. (**F**) Model-free seed-based correlation maps confirm spatial correspondence between resting-state fixation and task acquisitions using entire region seeds. Seed-based correlation maps from individual seed regions placed within networks DN-A and DN-B are displayed for three representative participants (P2, P6, and P12). Unlike Figure 3, which utilized a single vertex as a seed region, here the entire larger region serves as the seed region. These correlation maps, based exclusively on task-regressed functional connectivity, are plotted as z(r) with the color scale at the bottom. Black outlines show the boundaries of individual-specific networks estimated from independent resting-state fixation data within the same individual. White-filled regions with black outlines mark the seed region locations. The positions of the seed regions are moved across association zones between participants to demonstrate that the full distributed extent of DN-A and DN-B can be effectively generated from the many component regions. The network boundaries from resting-state fixation data align well with the spatial correlation properties of the task-regressed data establishing correspondence between resting-state fixation and task-regressed data. (**G**) Functional response effect sizes are larger for within-individual network estimates as compared to group estimates. Bar plots display mean z values for task responses to the Episodic Projection, Theory-of-Mind, and Sentence Processing task contrasts within the DN-A, DN-B and LANG networks, respectively. Task responses were measured in distributed association networks

defined either by a group-level prior ("Group") or within-individual network estimates ("Individual"). Rows show responses measured across a) the whole network, b) lateral prefrontal cortex (lPFC), c) medial prefrontal cortex (mPFC), and d) temporal cortex. Across all tasks and regions, individualized network estimates yield consistently higher mean z values than group-level estimates, particularly within anatomically complex cortical zones (lPFC, mPFC, and temporal cortex). Abbreviations: lPFC, lateral prefrontal cortex; mPFC, medial prefrontal cortex; SEM, standard error of the mean. DN-A, Default Network-A; DN-B, Default Network-B.

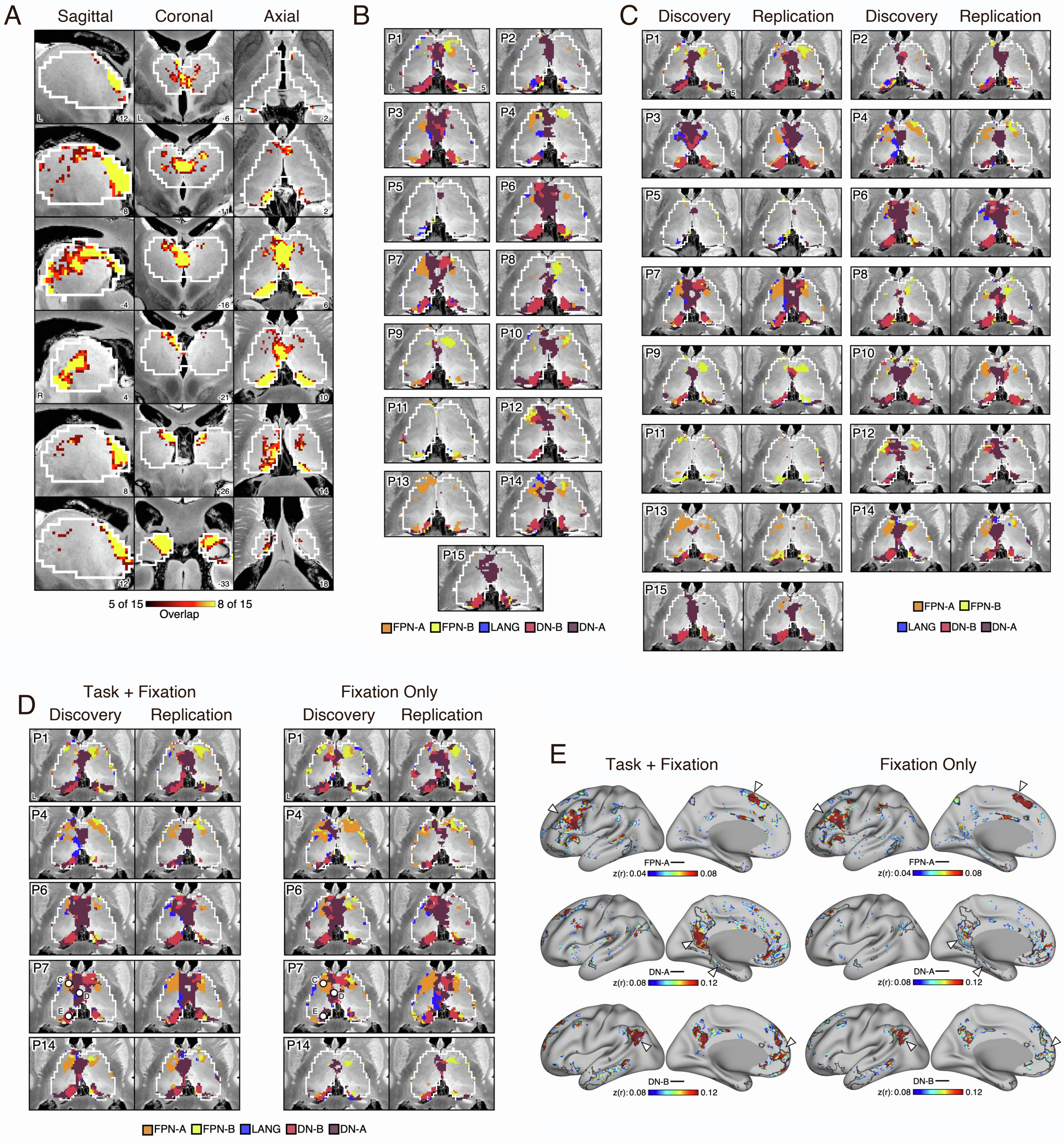

A
Sagittal
Coronal
Axial
5 of 15
Overlap
8 of 15
B
FPN-A FPN-B LANG DN-B DN-A
C
Discovery
Replication
FPN-A FPN-B LANG DN-B DN-A
D
Task + Fixation
Fixation Only
Discovery
Replication
FPN-A FPN-B LANG DN-B DN-A
E
Task + Fixation
Fixation Only
FPN-A
z(r): 0.04
0.08
DN-A
z(r): 0.08
0.12
DN-B
z(r): 0.08
0.12

Figure S5

Figure S5. (Related to Figure 6) Combining resting-state fixation and task-regressed data reveals thalamic subregions associated with distinct higher-order cortical networks.

Panels (**A**) Spatial overlap of thalamic subregions associated with higher-order cortical association networks across participants. Sagittal, coronal, and axial views illustrate the spatial consistency of thalamic subregions assigned to five higher-order cortical association networks across all participants (FPN-A, FPN-B, LANG, DN-A, and DN-B). Overlap is computed across all participants, with the scale at the bottom indicating the number of participants sharing a given voxel assignment (yellow: high overlap; red: low overlap). Higher-order association networks were linked to the thalamic midline extending from the anterior nucleus through the mediodorsal nucleus to the medial pulvinar. White outlines delineate the anatomical boundaries of the thalamus. (**B**) Best estimates of thalamic association network subregions across individuals. Thalamic organization is visualized within each individual using pooled data aggregated across all available resting-state and task-regressed data to reveal the best estimates of the five distinct association networks. Note that the idiosyncratic anatomical details differ between individuals but are reliable within a person. Further note that the distinct association zones have a general topographic pattern with FPN-A and FPN-B positioned anteriorly, DN-A falling along an extended midline zone, and DN-B positioned along the posterior midline. In many cases, multiple disjointed anterior and posterior subregions are linked to the same network. (**C**) Thalamic subregions linked to higher-order association networks are reliable within individuals across independent datasets. Thalamic organization is visualized for independent Discovery and Replication datasets within each individual to reveal the estimated locations of the five distinct association network representation. Note that the idiosyncratic anatomical details differ between individuals but are reliable within each person. Further note that the distinct association zones have a general topographic pattern with FPN-A and FPN-B positioned anteriorly, DN-A falling along an extended midline zone, and DN-B positioned along the posterior midline. In many cases though, multiple disjointed anterior and posterior subregions are linked to the same network. (**D**) Thalamic subregion estimates across individuals using pooled task-regressed and resting-state fixation data versus only resting-state fixation data. Thalamic organization is visualized for independent Discovery and Replication datasets for five representative participants (P1, P4, P6, P7, P14) to reveal the estimated locations of the five distinct association network representations. For each participant, parcellations generated from combined task-regressed + resting-state fixation data (left) and

fixation-only data (right) are shown. Note that the idiosyncratic anatomical details differ between individuals but are reliable within a person, and the within-individual consistency is greater when pooled data are used. Traditional analysis using resting-state data reveals many of the features, but not all, and with less stability. The results are more robust and consistent within individuals when pooled data are used. (**E**) Cortical functional connectivity patterns are clearer when pooling all available task-based and resting-state fixation data. Cortical functional connectivity patterns are visualized on the inflated surface for the same three distinct thalamic seed regions used in the Figures 6 and S5D (from P7 as displayed by white circles in panel B). Functional connectivity patterns derived from resting-state fixation data alone (right) are less selective and miss key network-defining brain regions - for example, posterior midline of DN-A, medial prefrontal regions of DN-B – compared to those derived from all available task-regressed and resting-state fixation data (left). This visualization illustrates the benefit of pooling all available within-individual data to enhance the precision mapping of cortical networks linked to the thalamus. L, left; FPN-A, Frontoparietal Network-A; FPN-B, Frontoparietal Network-B; DN-A, Default Network-A; DN-B, Default Network-B; LANG, Language.

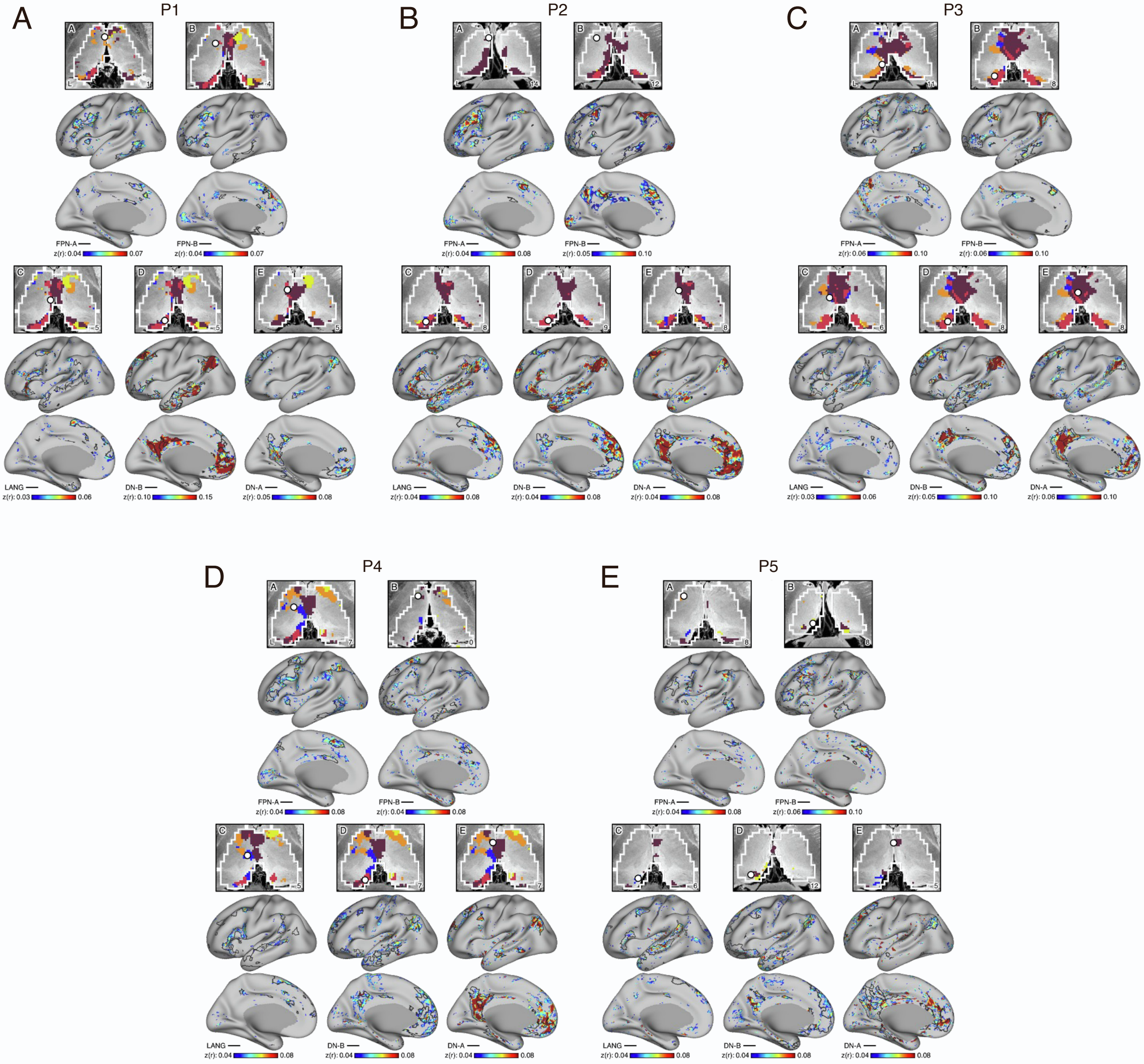

Figure S6

Figure S6. (Related to Figure 6) Juxtaposed thalamic subregions recapitulate distinct cortical association networks using pooled resting-state fixation and task-regressed data across many individuals (P1-P5, A-E).

Coronal sections display the thalamus within each participant, highlighting spatially segregated subregions associated with higher-order cortical association networks along the midline. White circles indicate the placement of seed regions for each association network: FPN-A, FPN-B, LANG, DN-B, and DN-A. Thalamic subregions were estimated from pooled within-individual resting-state fixation and task-regressed data. Model-free seed-region based correlation maps are projected onto the left cortical surface for each corresponding thalamic subregion. Each map reveals that thalamic seed region correlation on the cortical surface in relation to cortical network boundaries. The correspondence is impressive for most individuals for many of the networks. The correlation maps are plotted as z(r) with the color scale at the bottom. These results illustrate that closely juxtaposed yet spatially distinct thalamic subregions within an individual show functional connectivity to distinct cortical association networks, confirming the fine-grained topography of thalamo-cortical functional organization. Note that specificity was observed in many participants, with some individuals showing more distinct segregation of subregions between networks than others. L, left; FPN-A, Frontoparietal Network-A; FPN-B, Frontoparietal Network-B; DN-A, Default Network-A; DN-B, Default Network-B; LANG, Language.

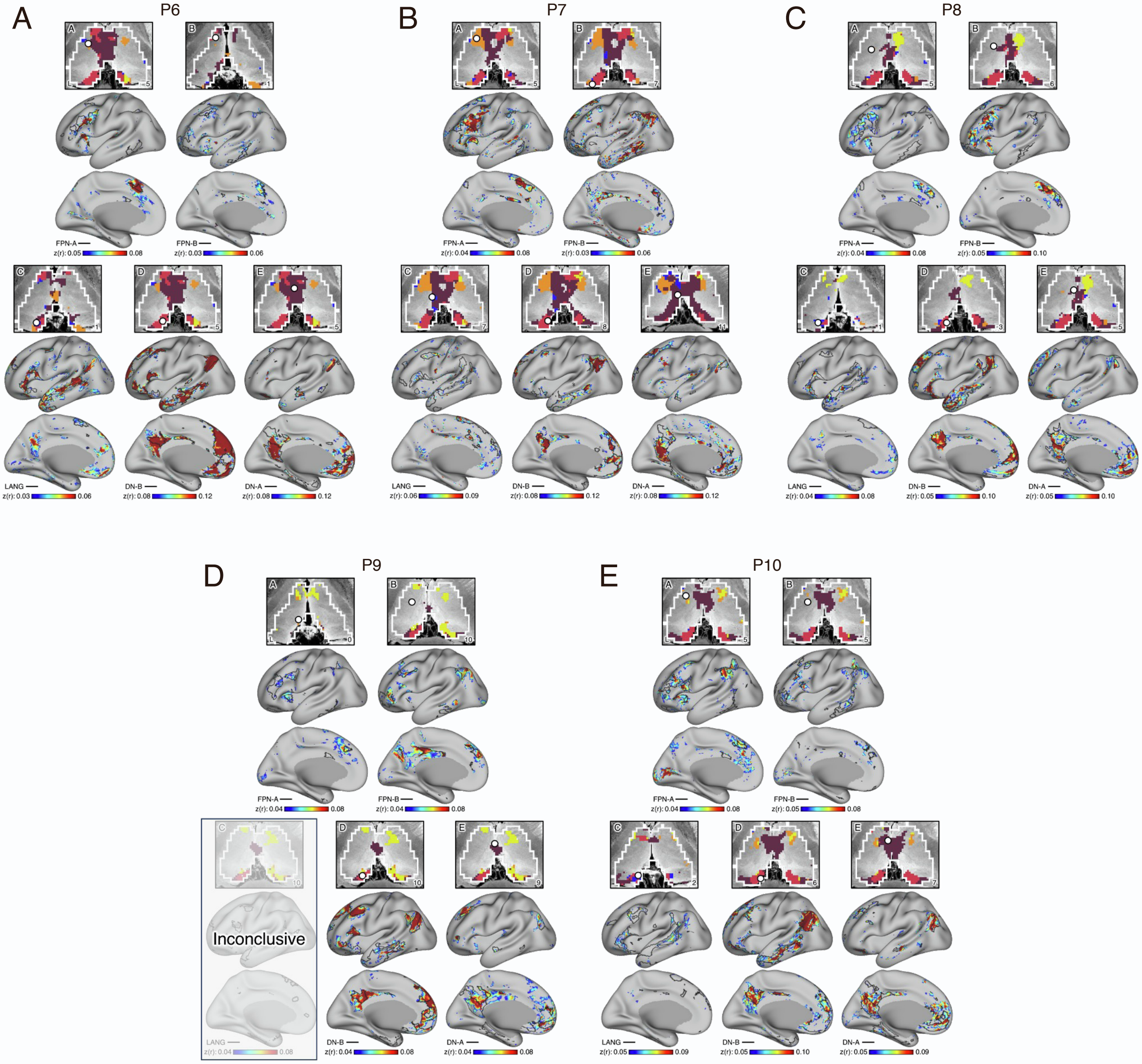
A
P6
FPN-A
z(r): 0.05 0.08
FPN-B
z(r): 0.03 0.06
LANG
z(r): 0.03 0.06
DN-B
z(r): 0.08 0.12
DN-A
z(r): 0.08 0.12
B
P7
FPN-A
z(r): 0.04 0.08
FPN-B
z(r): 0.03 0.06
LANG
z(r): 0.06 0.09
DN-B
z(r): 0.08 0.12
DN-A
z(r): 0.08 0.12
C
P8
FPN-A
z(r): 0.04 0.08
FPN-B
z(r): 0.05 0.10
LANG
z(r): 0.04 0.08
DN-B
z(r): 0.05 0.10
DN-A
z(r): 0.05 0.10
D
P9
FPN-A
z(r): 0.04 0.08
FPN-B
z(r): 0.04 0.08
Inconclusive
LANG
z(r): 0.04 0.08
DN-B
z(r): 0.04 0.08
DN-A
z(r): 0.04 0.08
E
P10
FPN-A
z(r): 0.04 0.08
FPN-B
z(r): 0.05 0.08
LANG
z(r): 0.05 0.09
DN-B
z(r): 0.05 0.10
DN-A
z(r): 0.05 0.09

Figure S7

Figure S7. (Related to Figure 6) Juxtaposed thalamic subregions recapitulate distinct cortical association networks using pooled resting-state fixation and task-regressed data across many individuals (P6-P10, A-E).

Coronal sections display the thalamus within each participant, highlighting spatially segregated subregions associated with higher-order cortical association networks along the midline. White circles indicate the placement of seed regions for each association network: FPN-A, FPN-B, LANG, DN-B, and DN-A. Thalamic subregions were estimated from pooled within-individual resting-state fixation and task-regressed data. Model-free seed-region based correlation maps are projected onto the left cortical surface for each corresponding thalamic subregion. Each map reveals that thalamic seed region correlation on the cortical surface in relation to cortical network boundaries. The correspondence is impressive for most individuals for many of the networks. The correlation maps are plotted as z(r) with the color scale at the bottom. These results illustrate that closely juxtaposed yet spatially distinct thalamic subregions within an individual show functional connectivity to distinct cortical association networks, confirming the fine-grained topography of thalamo-cortical functional organization. Note that specificity was observed in many participants, with some individuals showing more distinct segregation of subregions between networks than others. L, left; FPN-A, Frontoparietal Network-A; FPN-B, Frontoparietal Network-B; DN-A, Default Network-A; DN-B, Default Network-B; LANG, Language.

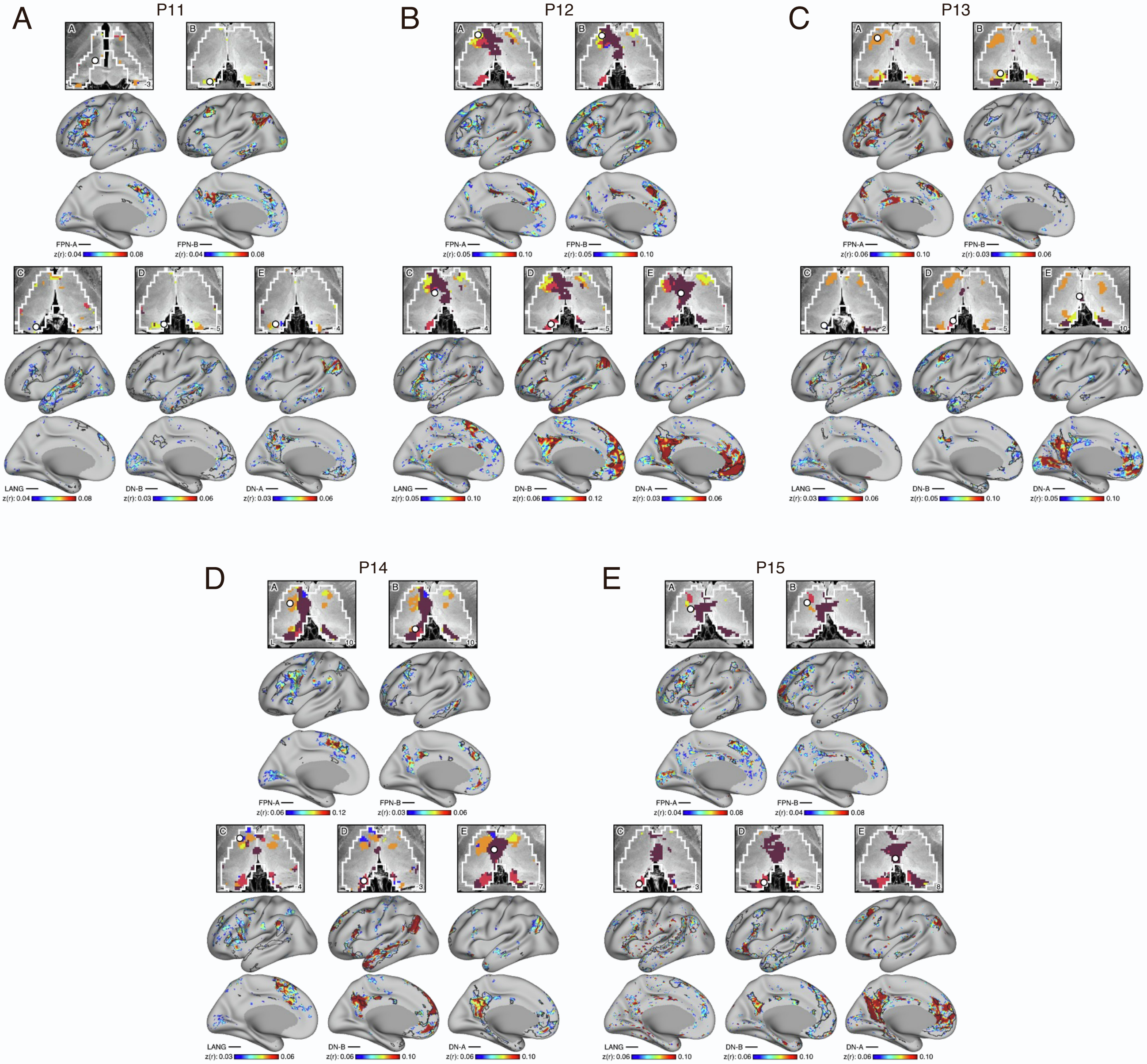

Figure S8

Figure S8. (Related to Figure 6) Juxtaposed thalamic subregions recapitulate distinct cortical association networks using pooled resting-state fixation and task-regressed data across many individuals (P11-P15, A-E).

Coronal sections display the thalamus within each participant, highlighting spatially segregated subregions associated with higher-order cortical association networks along the midline. White circles indicate the placement of seed regions for each association network: FPN-A, FPN-B, LANG, DN-B, and DN-A. Thalamic subregions were estimated from pooled within-individual resting-state fixation and task-regressed data. Model-free seed-region based correlation maps are projected onto the left cortical surface for each corresponding thalamic subregion. Each map reveals that thalamic seed region correlation on the cortical surface in relation to cortical network boundaries. The correspondence is impressive for most individuals for many of the networks. The correlation maps are plotted as z(r) with the color scale at the bottom. These results illustrate that closely juxtaposed yet spatially distinct thalamic subregions within an individual show functional connectivity to distinct cortical association networks, confirming the fine-grained topography of thalamo-cortical functional organization. Note that specificity was observed in many participants, with some individuals showing more distinct segregation of subregions between networks than others. L, left; FPN-A, Frontoparietal Network-A; FPN-B, Frontoparietal Network-B; DN-A, Default Network-A; DN-B, Default Network-B; LANG, Language.

Table S1. (Related to Figures 2-3) Fixation and task-regressed data used to generate precision maps of networks for each participant.

ID	FIX (runs)	MOT (runs)	VISME (runs)	ODDBALL (runs)	NBACK (runs)	SENT (runs)	ToM (runs)	EPRJ (runs)	Total Task Runs
P2	16	11	5	5	8	6	8	10	53
P3	19	10	5	4	8	6	7	8	48
P4	20	10	5	5	8	5	8	9	50
P6	21	12	5	5	8	6	8	10	54
P7	22	12	5	5	8	6	8	10	54
P8	21	12	5	5	8	6	8	10	54
P9	20	12	5	5	8	6	7	8	51
P12	24	24	5	4	8	11	8	10	70
P13	22	12	5	5	8	6	8	10	54
P14	19	9	5	5	8	6	8	10	51
P15	20	12	5	3	8	6	8	10	52

Notes: Numbers represent the number of runs. Participant IDs align with those in Du et al. [2]. Abbreviations: FIX = Resting-state Fixation; MOT = Motor; VISME = Visual Retinotopic Stimulation – Meridians / Eccentricity; ODDBALL = Visual Oddball Detection; NBACK = N-Back Working Memory; SENT = Sentence Processing; ToM = Theory-of-Mind; EPRJ = Episodic Projection.

Table S2. (Related to Figure 4) Alignment of task and fixation acquisitions for each participant in validity test of functional specificity.

	Data Used for Network Estimates							Task Response		
ID	FIX (min)	TASK (min)	FIX (runs)	MOT (runs)	ODDBALL (runs)	VISME (runs)	NBACK (runs)	SENT (runs)	ToM (runs)	EPRJ (runs)
P2	109	109	16	11	5	1	0	6	8	10
P3	130	128	19	10	4	5	3	6	7	8
P4	137	138	20	10	5	5	4	5	8	9
P6	144	143	21	12	5	5	2	6	8	10
P7	150	152	22	12	5	5	4	6	8	10
P8	144	143	21	12	5	5	2	6	8	10
P9	137	138	20	12	5	5	1	6	7	8
P12	164	163	24	17	4	5	0	11	8	10
P13	150	152	22	12	5	5	4	6	8	10
P14	130	131	19	9	5	5	4	6	8	10
P15	137	136	20	12	3	5	3	6	8	10

Notes: Amounts of data from task-regressed (MOT, ODDBALL, VISME and NBACK) runs and resting-state fixation (FIX) runs were matched in length within each individual to define networks. Independent task data (SENT, ToM and EPRJ) were used to examine the task response levels for the independently-defined networks. Numbers indicate the minutes or number of runs for each task, as labelled within the column. Participant IDs align with those in Du et al. [2]. Abbreviations: FIX = Resting-state Fixation; MOT = Motor; ODDBALL = Visual Oddball Detection; VISME = Visual Retinotopic Stimulation – Meridians / Eccentricity; NBACK = N-Back Working Memory; SENT = Sentence Processing; ToM = Theory-of-Mind; EPRJ = Episodic Projection.

Table S3. (Related to Figure 2) Overlap percentages of cortical vertices assigned to the same network across 15 networks between independent task and fixation datasets within the same individual.

Network	Overlap Percentages Mean (Range)	P2	P3	P4	P6	P7	P8	P9	P12	P13	P14	P15
SAL/PMN	82.6 (78.2-87.8)	79.6	84.5	82.3	83.0	78.2	87.8	80.9	81.8	82.3	82.3	85.3
CG-OP	83.1 (78.0-85.9)	80.2	81.3	85.9	83.4	82.9	84.3	85.1	84.5	83.1	78.0	85.6
FPN-A	79.5 (70.5-86.7)	73.1	80.4	86.7	86.5	84.3	81.5	70.5	80.5	85.2	70.6	75.1
FPN-B	80.5 (76.2-86.5)	78.6	78.7	76.2	86.5	83.9	81.0	80.8	81.5	81.6	79.0	78.1
DN-A	82.5 (78.4-85.9)	80.2	83.3	83.4	85.1	85.9	81.6	80.9	82.0	81.1	78.4	85.9
DN-B	81.4 (75.1-87.5)	75.1	80.2	80.0	87.5	84.2	84.1	77.7	83.8	78.9	80.6	82.8
LANG	77.1 (67.2-84.5)	67.5	72.0	81.4	78.9	80.6	82.8	67.2	74.4	84.5	78.5	80.6
dATN-A	73.8 (62.5-80.3)	72.1	75.7	78.4	79.2	72.2	80.3	69.9	76.6	71.5	62.5	73.1
dATN-B	69.7 (58.0-76.8)	76.8	63.7	71.6	76.7	69.8	76.1	58.0	72.2	71.4	59.6	71.4
PM-PPr	74.6 (59.7-87.2)	72.9	73.8	78.6	87.2	84.5	74.2	73.0	77.0	71.8	59.7	67.7
AUD	80.0 (68.9-85.4)	68.9	82.1	85.4	74.0	84.3	80.4	77.4	82.5	82.7	80.5	82.0
SMOT-A	86.1 (75.0-91.4)	87.1	87.8	90.0	80.2	87.4	91.4	89.1	86.0	75.0	83.2	90.1
SMOT-B	79.1 (40.2-88.4)	80.2	84.5	85.0	69.9	85.3	82.0	87.6	82.9	40.2	84.6	88.4
VIS-C	65.9 (48.4-79.9)	79.9	66.3	75.2	62.4	59.7	68.4	64.5	67.3	68.6	48.4	64.6
VIS-P	84.4 (77.1-90.5)	83.8	83.4	87.9	90.5	83.9	81.3	88.0	86.8	85.9	77.1	79.7

Notes: Numbers indicate the percentages of cortical vertices assigned to the same network between independent task and fixation datasets within the same individual. Participant IDs align with those in Du et al. [2]. Abbreviations: SMOT-A, Somatomotor-A; SMOT-B, Somatomotor-B; PM-PPr, Premotor-Posterior Parietal Rostral; CG-OP, Cingulo-Opercular; SAL / PMN, Salience / Parietal Memory Network; dATN-A, Dorsal Attention-A; dATN-B, Dorsal Attention-B; FPN-A, Frontoparietal Network-A; FPN-B, Frontoparietal Network-B; DN-A, Default Network-A; DN-B, Default Network-B; LANG, Language; VIS-C, Visual Central; VIS-P, Visual Peripheral; AUD, Auditory.

Supplemental References

1. Gratton, C., Laumann, T.O., Nielsen, A.N., Greene, D.J., Gordon, E.M., Gilmore, A.W., Nelson, S.M., Coalson, R.S., Snyder, A.Z., and Schlaggar, B.L. (2018). Functional brain networks are dominated by stable group and individual factors, not cognitive or daily variation. Neuron *98*, 439-452. https://doi.org/10.1016/j.neuron.2018.03.035.
2. Du, J., DiNicola, L.M., Angeli, P.A., Saadon-Grosman, N., Sun, W., Kaiser, S., Ladopoulou, J., Xue, A., Yeo, B.T., Eldaief, M.C., and Buckner, R.L. (2024). Organization of the human cerebral cortex estimated within individuals: Networks, global topography, and function. J. Neurophysiol. *131*, 1014-1082. https://doi.org/10.1152/jn.00308.2023.
